# Mapping the deformability of natural and designed cellulosomes in solution

**DOI:** 10.1186/s13068-022-02165-3

**Published:** 2022-06-20

**Authors:** Jonathan Dorival, Sarah Moraïs, Aurore Labourel, Bartosz Rozycki, Pierre-Andre Cazade, Jérôme Dabin, Eva Setter-Lamed, Itzhak Mizrahi, Damien Thompson, Aurélien Thureau, Edward A. Bayer, Mirjam Czjzek

**Affiliations:** 1 Integrative Biology of Marine Models (LBI2M), Station Biologique de Roscoff (SBR), Sorbonne Université, CNRS, 29680 Roscoff, Bretagne France; 2 Department of Biological Sciences, Vanderbilt University, Nashville, TN 37232 USA; 3 Department of Biomolecular Sciences, The Weizmann Institute of Science, 7610001 Rehovot, Israel; 4 Faculty of Natural Sciences, Ben-Gurion University of the Negev, 8499000 Beer-Sheva, Israel; 5 TBI, Université de Toulouse, CNRS, INRAE, INSA, Toulouse, France; 6 Institute of Physics, Polish Academy of Sciences, Al. Lotnikow 32/46, 02-668 Warsaw, Poland; 7 Department of Physics, Bernal Institute, University of Limerick, Limerick, Ireland; 8 Synchrotron SOLEIL, 91190 Saint Aubin, France

**Keywords:** Designer cellulosomes, Multi-enzyme complex, Scaffoldins, SAXS, Molecular modeling, Self-assembly, Bionanomachinery

## Abstract

**Background:**

Natural cellulosome multi-enzyme complexes, their components, and engineered ‘designer cellulosomes’ (DCs) promise an efficient means of breaking down cellulosic substrates into valuable biofuel products. Their broad uptake in biotechnology relies on boosting proximity-based synergy among the resident enzymes, but the modular architecture challenges structure determination and rational design.

**Results:**

We used small angle X-ray scattering combined with molecular modeling to study the solution structure of cellulosomal components. These include three dockerin-bearing cellulases with distinct substrate specificities, original scaffoldins from the human gut bacterium *Ruminococcus champanellensis* (ScaA, ScaH and ScaK) and a trivalent cohesin-bearing designer scaffoldin (Scaf20L), followed by cellulosomal complexes comprising these components, and the nonavalent fully loaded *Clostridium thermocellum* CipA in complex with Cel8A from the same bacterium. The size analysis of *R*_g_ and *D*_max_ values deduced from the scattering curves and corresponding molecular models highlight their variable aspects, depending on composition, size and spatial organization of the objects in solution.

**Conclusions:**

Our data quantifies variability of form and compactness of cellulosomal components in solution and confirms that this native plasticity may well be related to speciation with respect to the substrate that is targeted. By showing that scaffoldins or components display enhanced compactness compared to the free objects, we provide new routes to rationally enhance their stability and performance in their environment of action.

**Supplementary Information:**

The online version contains supplementary material available at 10.1186/s13068-022-02165-3.

## Background

Plant cell wall polysaccharides, mostly cellulose and hemicelluloses, are a major resource of carbon and energy [1], coveted by micro-organisms from all domains of life. Multi-component enzymatic complexes that can take different forms, depending on the nature and life style of the microbial organism using them, orchestrate the breakdown of these complex and recalcitrant components [2–4]. In particular, anaerobic bacteria have evolved a very sophisticated strategy to deconstruct recalcitrant plant cell wall components, which consists of an assortment of enzymes and auxiliary modules tethered together onto a more or less large scaffold protein, forming a macromolecular complex named cellulosome [5]. The synergistic effect of the multiple enzymes increases the degradation efficiency, for which the spatial arrangement between the enzymes in the cellulosome appears to be an essential key factor [6–9]. Recent genome mining has revealed a rich variety of such cellulosomal complexes, ranging from simple-architecture genomes that include a single scaffoldin protein to elaborate cellulosome assemblies that contain multiple scaffoldin proteins (ranging from 2 to 32 [10]). The scaffoldins, in turn, can display different degrees of complexity, ranging from 2 to 3 cohesin module-containing scaffoldins to those that can attach up to fifteen enzymes at a time [11]. Inspired by natures’ Lego-like manipulating of these complexes, employing them such to adapt to different lifestyles or substrates [12], recent efforts have also focused on conceiving and studying so-called ‘designer cellulosomes’ [13–17].

Rational design of cellulosome complexes requires in-depth knowledge of the synergistic structure/function relationship exhibited by its components. It is thus crucial to map the structural arrangement of cellulosomes at the molecular level to understand the structural basis for their high efficiency, but these efforts are hampered by the high proportion of unstructured linkers, their large size, and the intrinsic flexibility of scaffoldins [18, 19]. Although the structures of individual dockerins, cohesins, scaffoldin segments, carbohydrate binding modules (CBMs) and enzymes have been solved by crystallography and NMR [20–25] and are accessible, little is known about the global organization of an entire cellulosome or even a complete scaffoldin.

Recently, small-angle X-ray scattering (SAXS) and cryo-electronic microscopy (cryo-EM) were used to assess the structure of cellulosomal components in near-in-vivo conditions [26]. Early microscopic studies had already revealed the flexibility of the cellulosome, which grants its plasticity with the ability to adopt a tight or loose conformation depending on conditions [27]. Subsequently, a “dissect and build” strategy was adopted to study small portions of the scaffoldin CipA from *Clostridium thermocellum* [21, 22, 26]. This allowed the piecemeal reconstitution of 75% of the full-length protein [26]. The cryo-EM studies of a mini-cellulosome, comprising cohesins 3–5 of CipA bound to three copies of Cel8A, revealed the presence of both a compact and a more open and flexible conformation [28]. In both cases, the catalytic domains are projected, alternatingly, in opposite directions. García-Alvarez et al. also determined that linkers between two consecutive cohesins exhibit more flexibility than the linker between the enzymes and their dockerin. Furthermore, a combined SAXS and biochemical study of two consecutive cohesins joined by an engineered linker revealed that the length and the flexibility of the linker did not significantly affect the synergy between the enzymes bound to the cohesins [29]. To date, it has not proved possible to decipher structural arrangements in a more complete and natural scaffoldin, composed of more than three cohesins.

Computational biology is an emerging and complementary method, which allows prediction of the dynamics of cellulosomal components [30], their influence on the catalytic active site [31] or the behavior of cellulosomal modules in contact with substrates of different nature [32]. In our current study, we combined experiment and simulations to complete some important “missing pieces” of the scaffoldin structural map. First, we analyze small-sized scaffoldins from *Ruminococcus champanellensis* that, unusually, contain alongside the cohesins either X-modules or catalytic domains within the primary sequence of the scaffoldin. Second, we combine SAXS, homology modeling, coarse-grained (CG) molecular modeling and atomistic molecular dynamic simulations to characterize the structure and flexibility of an efficient “designer cellulosome (DC)” [13]. We thus adopted the ‘dissect-and-build’ strategy to study a DC composed of three chimeric cohesins, as they interact with three partner enzymes. Finally, we attempt to investigate the global shape in solution of an intact, full-length, nonavalent wild-type scaffoldin, CipA from *C. thermocellum*, alone and in complex with nine copies of the wild-type *C. thermocellum* Cel8A-*t* enzyme.

## Results

**Fig. 1:**
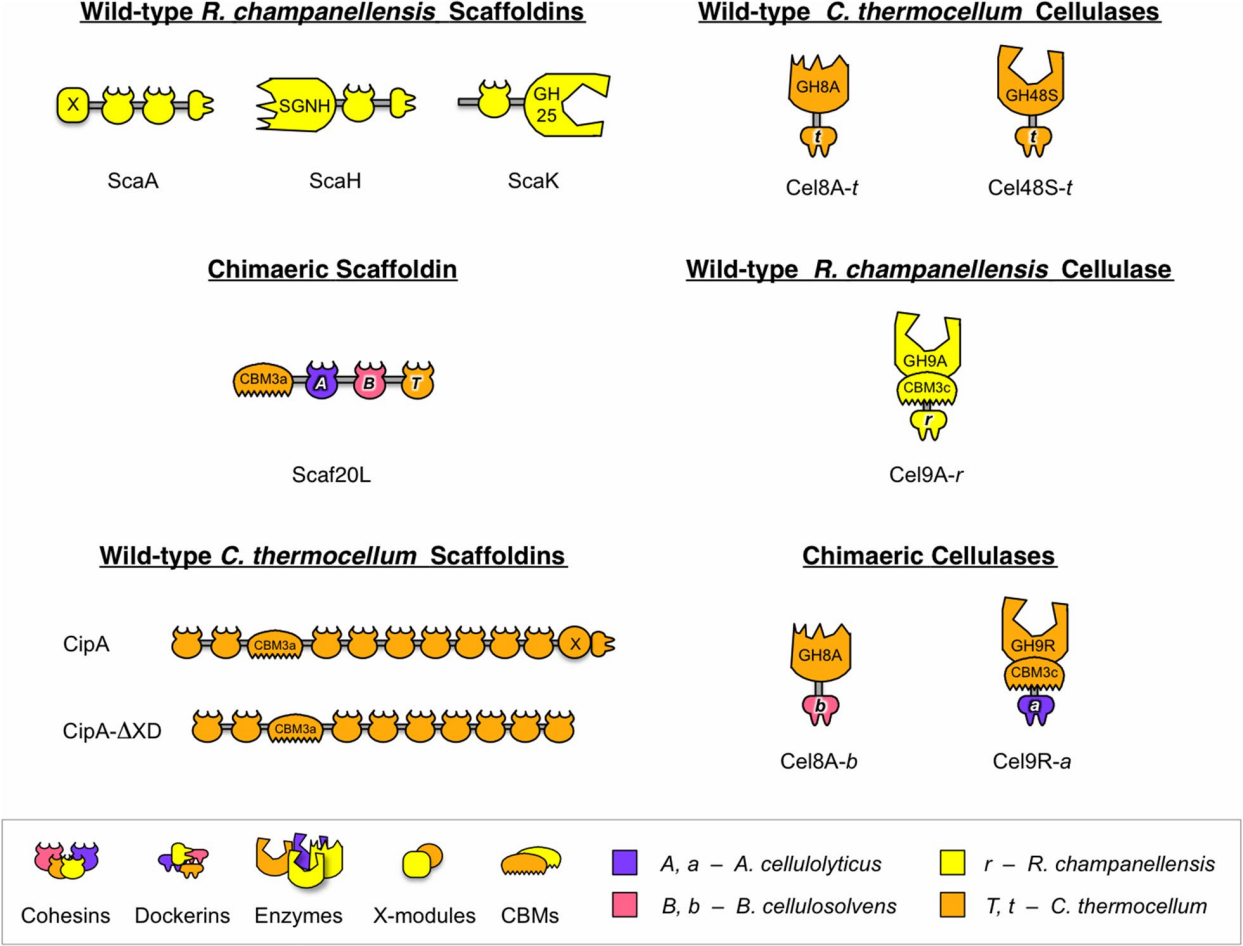
Schematic representation of cellulosomal components, enzymes and scaffoldins that were studied in this work. See Additional file 1: Table S2 for details and sequences

To date, no crystallographic structure of an entire cellulosome has been successfully solved, most probably due to the difficulty of obtaining crystals, owing to the inherent flexibility of the linker regions in the scaffoldin, their glycosylation in most species, the heterogeneity in enzyme content and disposition, and the dual mode of binding [26, 33–35]. Thus, in the present work, we employed a combination of SAXS and molecular modeling to study both natural cellulosome components and a designer cellulosome, composed of recombinant, chimeric components (Fig. 1). As SAXS is a powerful method to study the shape of large and flexible proteins directly in solution, by combining SAXS with homology modeling and molecular simulations (coarse-grained and atomistic), we can generate several physically realistic models, which can be further refined against the experimental data. The aim of this work was to better understand how the intrinsic flexibility and the structural heterogeneity of cellulosome systems vary with changing composition and constituents, by studying several natural cellulosomal scaffoldins, which display various sizes, together with one chimeric DC. To this end, we targeted three different small scaffoldins (ScaA, ScaH, ScaK) from the human gut bacterium *Ruminococcus champanellensis*, a chimeric designer cellulosome composed of a trivalent scaffoldin Scaf20L together with two chimeric enzymes and one wild-type enzyme, and a large natural scaffoldin, namely, CipA from *Clostridium thermocellum* [14, 36, 37]. All of the latter target proteins were produced recombinantly. See Fig. 1 for a schematic representation of the scaffoldins and enzymes used in this work.

Following the ‘dissect and build’ strategy [38], we first collected scattering curves for individual dockerin-containing enzymes that were subsequently integrated into the complex DCs, with the aim of comparing the *D*_max_ and *R*_g_ values before and after incorporation.

### SAXS analyses of individual modules, enzymes and components

#### Cel8A-b, Cel9A-a, Cel48S-t

**Fig. 2:**
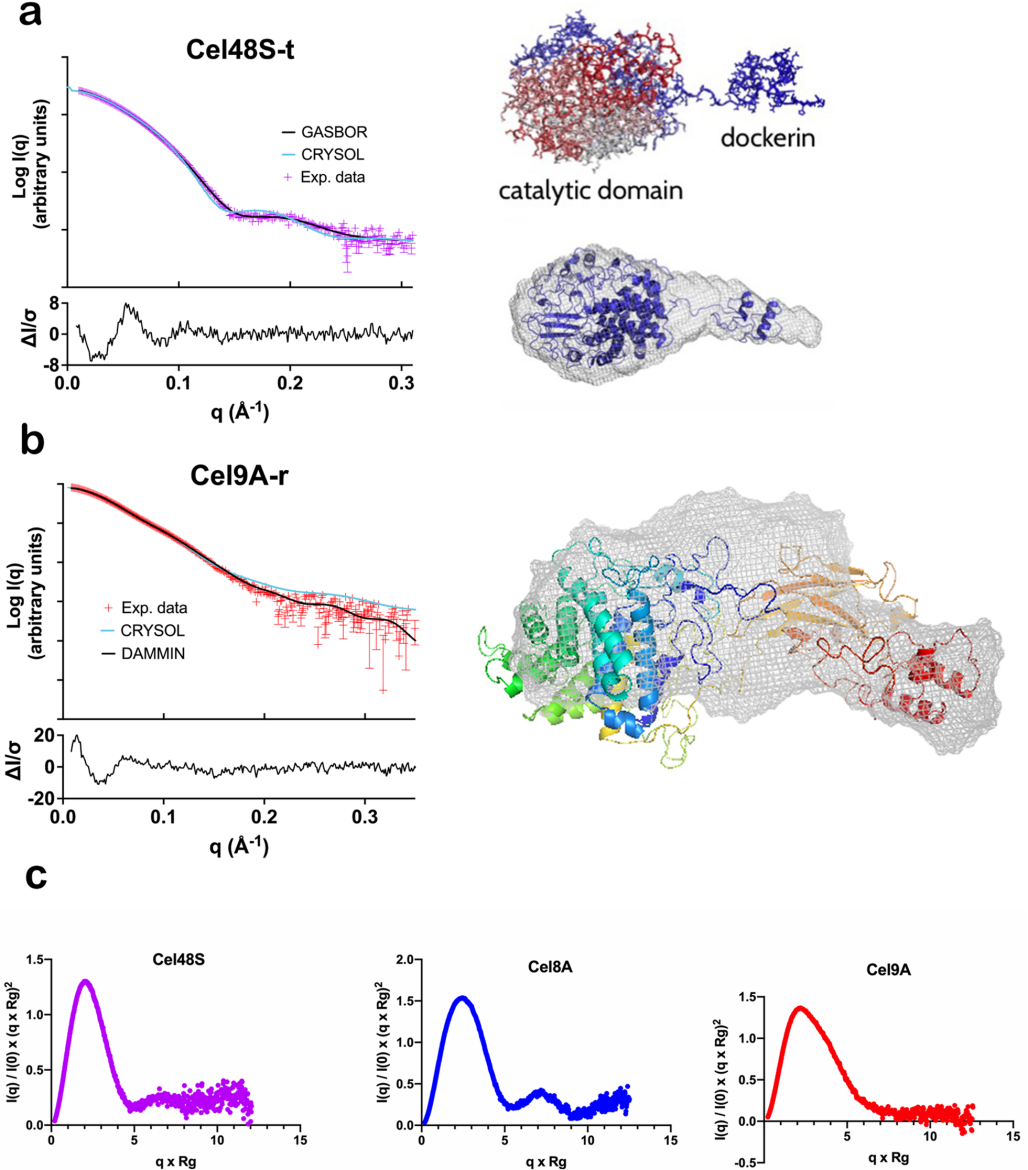
GASBOR/DAMMIN-Fit and “solution structure” images of the individual enzymes. **a** Cel48S-*t;* left panel: comparison between the experimental SAXS data (red points) and the scattering intensity I(q) of the Cel48S-*t* envelope obtained by GASBOR (black line) [72], and with that of the constructed structural model using CRYSOL (light blue line) [40]; residuals are illustrated for the envelope calculation; right panel (top): SAXS-derived structural model of the full-length cellulase Cel48S-*t*. The catalytic domain is separated from the dockerin domain by a linker in an extended conformation; right panel (bottom): comparison between the SAXS-derived structural model (blue) and the molecular envelope generated using GASBOR (transparent grey) [73]. **b** Cel9A-*r*; left panel: experimental curve fitted by DAMMIN (black line) [44] and CRYSOL (light blue line) [40] using the coarse grain model of the full length enzyme; residuals are illustrated for the DAMMIN fit; right panel: superimposition of the coarse grain model onto the most representative DAMMIN envelope. **c** Representation of the scattering curves as Kratky plots for the three individual enzymes Cel48S*-t*, Cel9A*-r* and Cel8A*-b*, indicating their mainly compact and globular shape

The resulting experimental curves for the individual elements are represented in Fig. 2, and *R*_g_ and *D*_max_ values are given in Table 1 (additional values are given in Additional file 1: Table S1. Guinier plots are given in Additional file 2: Fig. S1). For the chimeric Cel8A-*b* and wild-type Cel48S-*t,* the data are in agreement with previous SAXS studies on similar objects [20, 22], with *D*_max_ and *R*_g_ proportional to molecular weight, showing that the linkers between the catalytic modules of Cel8A-*b* and Cel48S-*t* and their respective dockerins, both of which contain 19 residues, are rather extended. The pair distribution of the data acquired on Cel48S-*t* from *C. thermocellum* alone indicates a *D*_max_ of 148 ± 4 Å and shows that the wild-type enzyme is a globular protein with an extended extremity. A homology model refined by coarse-grained simulations of Cel48S-*t*, based on the structure of its catalytic domain (PDB = 1L1Y) [39], was created and fitted to the SAXS data using CRYSOL [40], as illustrated in Fig. 2a. The model that fits the SAXS data best (χ^2^ = 1.29) was selected from a pool of 2 × 10^5^ structural models of the full-length Cel48S-*t* cellulase, highlighting the extended linker.

**Table 1:** Experimental SAXS parameters derived from the scattering curves of the various scaffoldins, components and complexes

Construct	Organism	*R*_g_ (Å)	*D*_max_ (Å)	*M*_w_ (kDa)	*χ*^2^ of fit
Scaffoldins and components					
ScaA	*R. champanellensis*	60.4 ± 0.2	282 ± 11	68.7	3.1
X module of ScaA	*R. champanellensis*	21.6 ± 0.1	91 ± 5	23.2	3.7
ScaH	*R. champanellensis*	55.8 ± 0.2	230 ± 15	55.1	3.1
SGNH module of ScaH	*R. champanellensis*	27.1 ± 0.6	103 ± 5	29.3	2.6
ScaK	*R. champanellensis*	44.9 ± 0.2	184 ± 6	52.3	5.3
Scaf20L	Chimeric scaffoldin: CBM and cohesin from *C. thermocellum,* cohesins from *A. cellulolyticus* and *B. cellulosolvens*	66.3 ± 0.4	262 ± 10	75.3	1.1
Enzymes					
Cel48S-*t*	Wild-type *GH48S* and *dockerin* from *C. thermocellum*	34.2 ± 0.1	148 ± 4	81.6	1.3
Cel8A-*b*	Chimeric enzyme: GH8 from *C. thermocellum,* dockerin from *B. cellulosolvens*	29.9 ± 0.3	118 ± 3	51.6	8.3
Cel9A-*r*	Wild-type enzyme, GH9-CBM3c and dockerin from *R. champanellensis*	35.2 ± 0.1	110 ± 3	91.8	5.5
DC complexes					
Scaf20L + Cel8A-*b*	Chimeric scaffoldin and chimaeric enzyme	64.3 ± 0.3	251 ± 8	126.9	1.1
Scaf20L + Cel9R-*a* + Cel8A-*b* + Cel48S-*t*	Chimeric scaffoldin and chimaeric and wild-type enzymes from *C. thermocellum* bearing dockerins that match the cohesins of Scaf20L	90.9 ± 0.5	305 ± 15	300.3	1.3
CipA and complex					
CipA	Wild-type scaffoldin from *C. thermocellum*	157 ± 1.6	530 ± 20	198.1	ND
CipA-∆XD + Cel8A-*t*	Truncated scaffoldin and wild-type enzyme from *C. thermocellum*	151 ± 1.7	497 ± 18	632.2	ND
CipA + Cel8A-*t*	Wild-type scaffoldin and wild-type enzyme from *C. thermocellum*	170.1 ± 1.2	575 ± 20	651.7	ND

Handling the protein sample of the Cel9R-*a* chimeric protein (GH and CBM3c from *C. thermocellum* and its wild-type dockerin replaced by a dockerin from *A. cellulolyticus)* in concentrations needed for SAXS measurements proved challenging. We thus analyzed instead a homologous wild-type protein from *R. champanellensis,* termed Cel9A-*r* that has the exact same modular composition (see Fig. 1 and Additional file 1: Table S2). Interestingly, the result for Cel9A-*r*, that also contains a CBM3c module tightly tethered to the catalytic module (Fig. 2b), is an exception to the proportionality of *D*_max_ and *R*_g_ of dockerin-containing enzymes vs. their mass (Table 1), since the overall shape is more compact than Cel8A-*b* or Cel48S-*t* as illustrated by the Kratky-plots (Fig. 2c), even though Cel9A-*r* is larger and has a longer linker region (29 residues). An atomic model could be built, since crystal structures for all individual modules of Cel9A-*r* are available, and, using CRYSOL [40], the compact form of the model was calculated to fit the experimental scattering curve with a poor χ^2^ of 4.1. The flexibility of the linker was assessed by MD-simulations, and fitting of these models using the EROS method [41] revealed that an ensemble of structures fits the experimental curve better than individual structures (χ^2^ of 3.0; Additional file 3: Fig. S2).

### SAXS measurements of scaffoldin variants

For all three scaffoldin variants, ScaA, ScaH and ScaK, no crystallographic structures are available, we have thus analyzed and compared their compactness by Kratky plots [42, 43] and ab initio envelope calculations using DAMMIN [44]. To verify their compact and globular character, we have collected SAXS curves for the individual X-module of ScaA and of SGNH present in ScaH (see Additional file 1 and Additional file 4: Fig. S3).

**Fig. 3:**
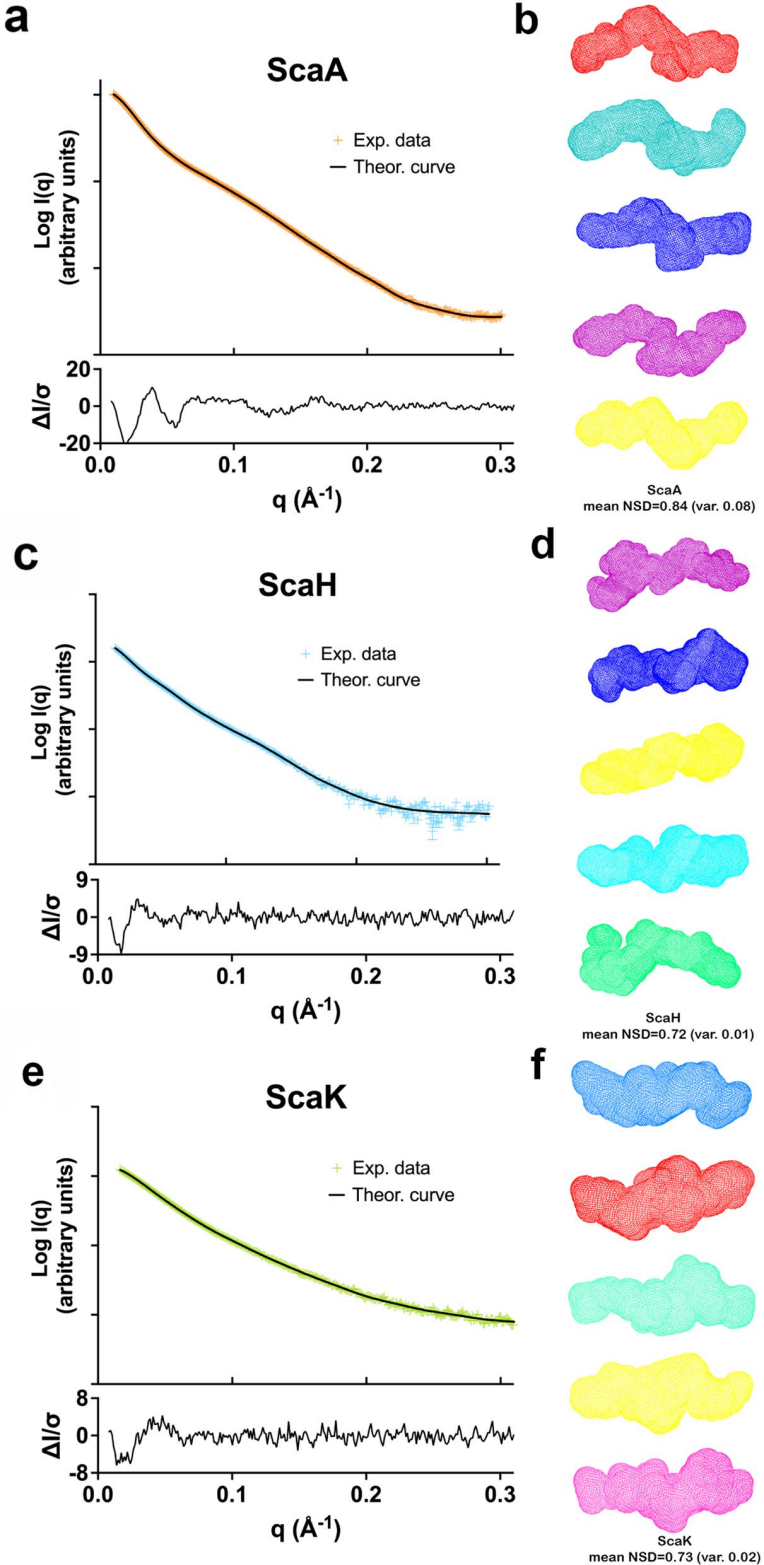
Experimental scattering of the scaffoldin proteins ScaA, ScaH and ScaK and their analyses using ab initio envelope calculations. **a** Experimental scattering curve (orange points) of ScaA fitted by DAMMIN (black line) [73]; **b** ScaA; representation of 5 examples out of 10 fitted DAMMIN envelopes (colored red, cyan, blue, magenta and yellow); the overall mean normalized spatial discrepancy (NSD) calculated with DAMAVER for the 10 independent envelopes is 0.84 with a variation of 0.08, a value that indicates conservation of the shapes. All envelopes show similar kinks, indicating and coherent with the modular composition of the scaffoldin. **c**. Experimental scattering curve (light blue points) of ScaH fitted by DAMMIN (black line) [73]. **d** ScaH; representation of 5 examples out of 10 fitted DAMMIN envelopes (colored magenta, blue, yellow, cyan and green); the overall mean NSD calculated with DAMAVER for the 10 independent envelopes is 0.72 with a variation of 0.01, a value that indicates conservation of the shapes. The various envelopes highlight the modular composition of the scaffoldin, but locating individual modules within the shape is not possible. **e** ScaK; Experimental scattering curve of ScaK (green points) fitted by DAMMIN (black line) [73]. **f** ScaK; representation of 5 examples out of 10 fitted DAMMIN envelopes (colored blue, red, green, yellow and magenta); the overall mean NSD calculated with DAMAVER for the 10 independent envelopes is 0.73 with a variation of 0.02, a value that indicates conservation of the shapes. In coherence with *R*_g_ and *D*_max_ values and the Kratky plot of scattering originating from ScaK, the envelopes show a more compact shape of this scaffoldin

**Fig. 4:**
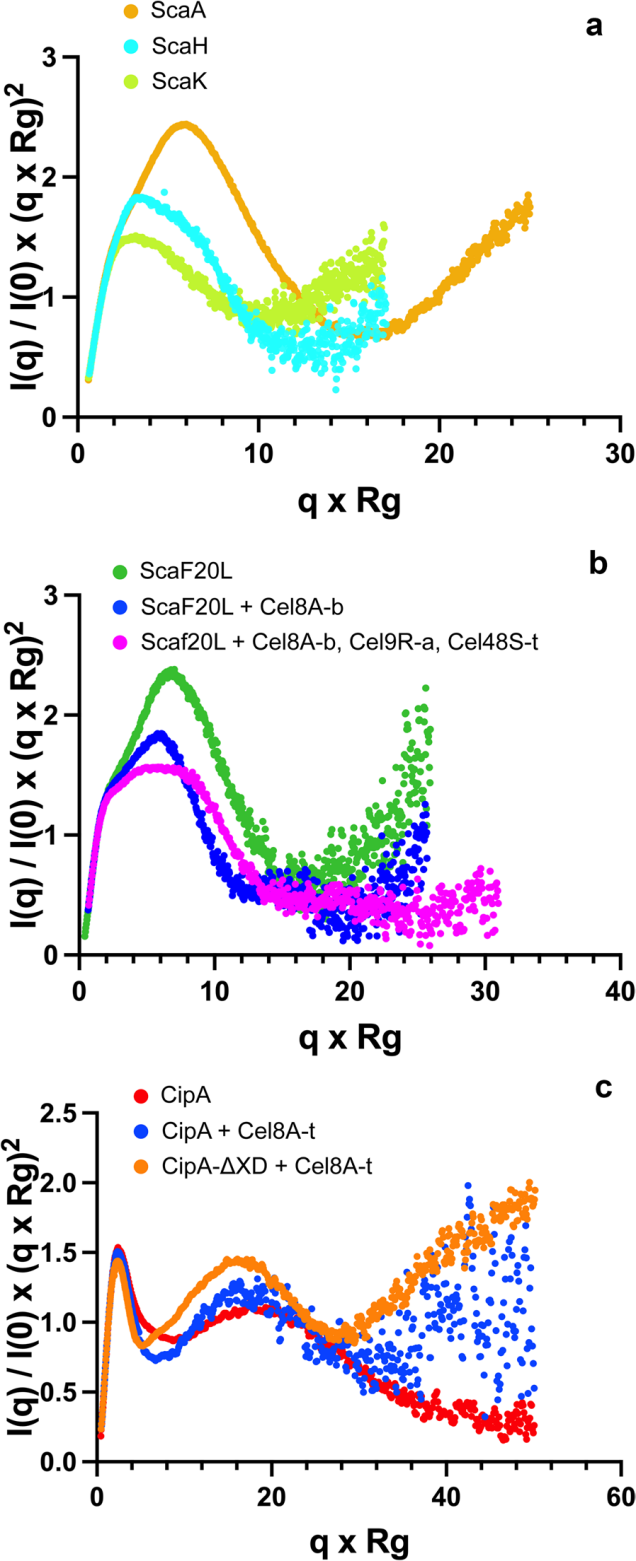
Representation and superimposition as normalized Kratky plots of various scattering curves obtained for related objects **a** normalized Kratky plots for ScaA, ScaH and ScaK. **b** normalized Kratky plots for Scaf20L alone, Scaf20L in complex with Cel8A*-b* and Scaf20L in complex with Cel8A*-b*, Cel9R*-a* and Cel48S*-t*. **c** normalized Kratky plots for CipA, CipA + Cel8A*-t* and CipA-ΔXD + Cel8A*-t*

ScaA is a 68-kDa protein, which is composed of an X-module, two cohesins and a dockerin (Fig. 1, Additional file 1: Table S2). SAXS data of good quality (Additional file 5: Fig. S4a) were acquired for this construct (Fig. 3a, orange curve), which allowed determination of *R*_g_ as 60.9 Å and *D*_max_ = 282 ± 11 Å (Table 1). As is highlighted by the Kratky plot (Fig. 4a, orange curve), the obtained scattering curve is consistent with a non-globular, elongated and partially flexible protein. We then calculated ab initio envelopes in multiple independent runs using DAMMIN [44] as described in the Methods section. Despite some apparent partial unfolded parts, identified in the Kratky plot at high *Q* values, the normalized spatial discrepancy (NSD) obtained over 10 calculations is 0.84 < 1, which indicates that the shape of the envelopes is rather conserved. All shapes display the same kinks (Fig. 3b) consistent with the presence of four distinct modules, but the relative orientation of the individual modules remains ambiguous.

ScaH (Fig. 1, Additional file 1: Table S2) is composed of a catalytic domain, consisting of an SGNH homologue of a lipase or an esterase, a cohesin and a dockerin. First, the Guinier approximation of the SAXS data allowed us to determine an *R*_g_ value of 56.4 Å (Fig. 3c, light blue curve). The Kratky plot (Fig. 4a, cyan curve) is typical for a scaffoldin, meaning that ScaH is a non-globular, extended and multi-domain protein (Tables 1 and Additional file 5: Fig. S4). The envelopes that best fit the experimental curve (Fig. 3d) calculated with DAMMIN [44] are in agreement with this multi-domain architecture. Likewise ScaA, the normalized spatial discrepancy (NSD) obtained for 10 envelope calculations is 0.72 < 1 and consistent with conserved shapes (Fig. 3d).

Similar to ScaH, the scaffoldin ScaK also possesses an additional catalytic domain in the primary sequence, which belongs to GH25. Besides the GH25 domain, ScaK is composed of only one cohesin and it lacks a dockerin. An unidentified 103-residue stretch precedes the cohesin at the N terminus (Additional file 1: Table S2), which could indicate the presence of an additional small domain or module. The SAXS data (Fig. 3e, light green curve) indicate *R*_g_ of 45 Å and *D*_max_ of 184 ± 6 Å, which is significantly more globular and compact than ScaH. This is also illustrated by the Kratky-plot (Fig. 4a, light green curve), where the maximum of the bell shape is shifted to lower values as compared to ScaH and notably ScaA. Repeated DAMMIN [44] calculations yielded conserved envelopes (NSD = 0.73) that fit the experimental curve, as exemplified in Fig. 3e, with similar *χ*^2^ values, the best being 3.1. Nevertheless, these SAXS data measured on ScaK do not allow us to identify the relative positions of the domains within the molecular envelopes with confidence, even if a more globular shape in the middle of most envelopes would suggest that the GH25 adopts a central position (Fig. 3f).

### SAXS analyses of a designer cellulosome Scaf20L

To facilitate the incorporation of catalytic subunits onto the scaffoldin, we designed a trivalent chimeric scaffoldin, composed of three cohesins from different organisms and a cellulose-binding CBM (Fig. 1). These include the third cohesin of ScaB from *B. cellulosolvens*, the third cohesin of ScaC from *A. cellulolyticus*, the second cohesin and the CBM3a of CipA from *C. thermocellum*. In addition, we prepared three cellulosomal enzymes, which contain three different types of *C. thermocellum*-based catalytic domains connected to a dockerin that matches the specificity of the Scaf20L cohesins. Thus, the wild-type *C. thermocellum* dockerins of endoglucanase Cel8A and processive endoglucanase Cel9R were replaced with dockerins from *B. cellulosolvens* and *A. cellulolyticus,* respectively, to produce the corresponding chimeric enzymes. The wild-type *C. thermocellum* exoglucanase Cel48S*-t* was used with its native dockerin intact. In this way, each enzyme displays a dockerin complementary to a single cohesin in the chimeric Scaf20L scaffoldin, thus avoiding unwanted random or unspecific assembly that would otherwise occur (Fig. 1). This strategy ensures the specificity of each interaction and allows production of a monodisperse solution for the complex, which is required for SAXS. Such trifunctional designer cellulosomes have been reported to exhibit enhanced performance relative to equimolar mixtures of the free enzyme components [45].

#### Scaf20L alone

The SAXS analysis of the small chimeric Scaf20L scaffoldin turned out to be more complicated than expected. The methods based on the light scattering are very sensitive to the presence of several different species in solution. The *D*_max_ value of this construct was difficult to establish without ambiguity. However, *D*_max_ of 262 ± 10 Å gave the best fit and the most realistic distance distribution function (Additional file 6: Fig. S5c, green curve). This ambiguity of the *D*_max_ value already provided us insight about the flexibility of the protein and may indicate the presence of several conformers in solutions. The Kratky plot (Fig. 4b, green curve) confirmed that Scaf20L is a non-globular and partially flexible protein. Furthermore, shape calculations show two majority envelopes: an “extended” one, which is 40 Å longer than an alternative more compact shape of about 200 Å in length.

**Fig. 5:**
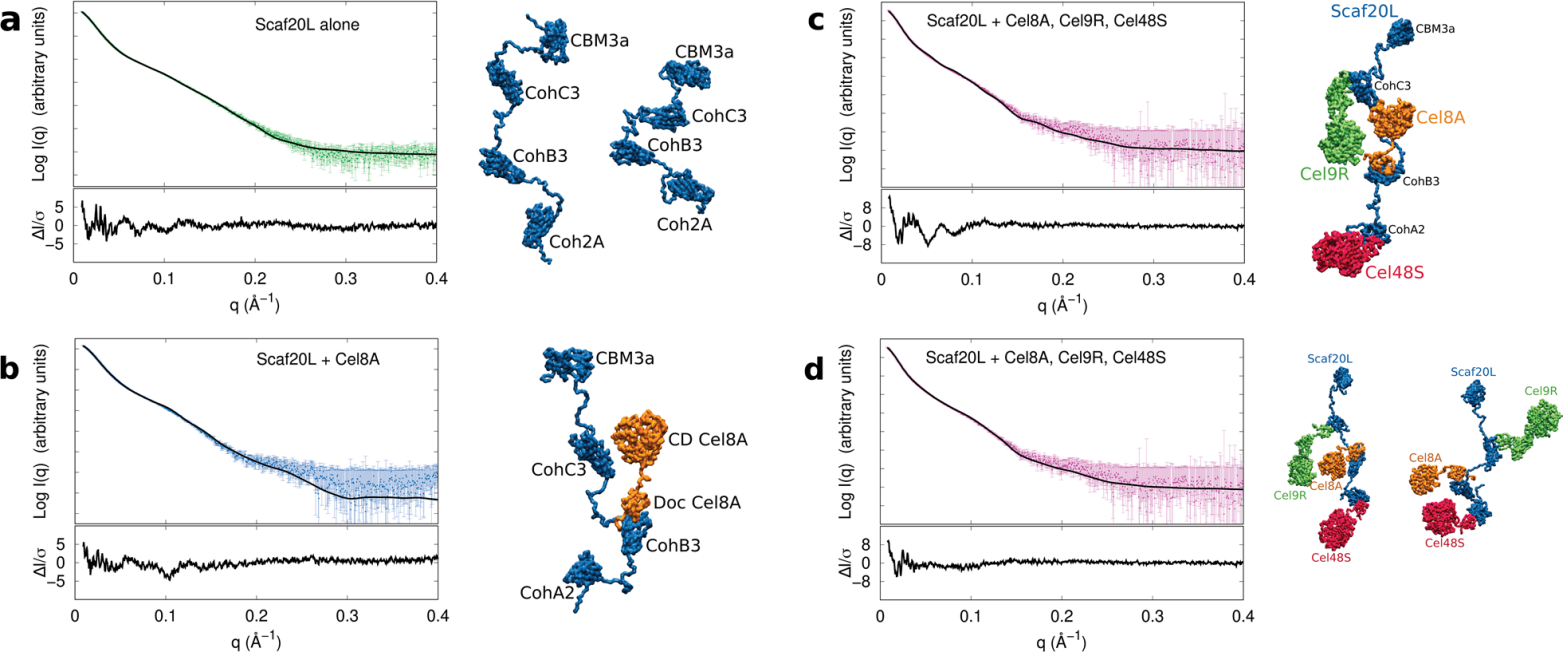
Molecular modeling to fit the experimental SAXS curves of various DCs based on Scaf20L. **a** Left panel: experimental scattering curve of Scaf20L alone. The black line represents the best fit (mixture of extended and compact forms). Right panel: two structural models of scaffoldin Scaf20L that jointly fit the experimental SAXS data. The four domains of Scaf20L are indicated: the second cohesin of CipA from *C. thermocellum* (Coh2A), the third cohesin of ScaB from *B. cellulosolvens* (CohB3), the third cohesin of ScaC from *A. cellulolyticus* (CohC3) and the cellulose binding module of CipA from *C. thermocellum* (CBM3a). Note that none of the two structural models separately fits the experimental SAXS data. However, the two models taken together with equal statistical weights fit the SAXS data very well, see the black curve in **a**. **b** Left panel: experimental scattering curve of Scaf20L In complex with Cel8A-*b*. The black line represents the best fit obtained by the structural model presented in the right panel. Right panel: SAXS-derived structural model of the Scaf20L:Cel8A-*b* protein complex. Cellulase Cel8A-*b* is shown in orange, where its *C. thermocellum* catalytic domain (CD Cel8A) and *B. cellulosolvens* dockerin (Doc Cel8A) are labelled. The scaffoldin Scaf20L is shown in blue, with its cohesin and CBM modules labelled as in Fig. 5a. The disordered linkers adopt extended conformations. Note: a single Cel8A-*b* enzyme component interacts selectively via its *B. cellulosolvens* dockerin with the matching cohesin (CohB3) of Scaf20L and fails to interact with the other two non-matching cohesins. **c** Left panel: experimental scattering curve of Scaf20L in complex with 3 enzymes. The black line represents the best fit obtained by a single structural model presented in the right panel (*χ*^2^ = 1.87). Right panel: detail of the SAXS-derived structural model of the Scaf20L-based complex with 3 enzymes. Scaffoldin Scaf20L is shown in blue, Cel48S-*t* in red, Cel9R-*a* in green, and Cel8A-*b* in orange. In this structure, the disordered linkers in Scaf20L adopt the most extended conformations. **d** Left panel: experimental scattering curve of Scaf20L in complex with 3 enzymes. The black line represents the best fit obtained by a mixture of the structural models presented in the right panel (*χ*^2^ = 1.25). Right panel: two models of Scaf20L in complex with 3 enzymes. The color code is the same as in right panel of Fig. 5c. Neither of the two models separately fits the experimental SAXS data as well as the two models taken together with equal statistical weights

In the pool of 2 × 10^5^ structural models of the scaffoldin Scaf20L, calculated as described in the methods section, we identified several models consistent with the experimental SAXS data (1.8 < *χ*^2^ < 2). However, a much better fit to the SAXS data (*χ*^2^ = 1.04; Fig. 5a, left panel) was found for a set of two structural models taken with equal statistical weights (Fig. 5a, models I and II). One of the models corresponds to an extended conformation, while the other one represents a compact conformation of Scaf20L. Our analysis indicates that these two models together represent the minimal ensemble of the Scaf20L conformations in solution.

#### Scaf20L in complex with Cel8A-b (monovalent DC complex)

We next investigated the chimeric Scaf20L scaffoldin in complex with cellulase Cel8A-*b* (Fig. 5b, Additional file 6: Fig. S5 blue line). From the pool of 2 × 10^5^ structural models calculated for this composition, we selected one model of the Scaf20L:Cel8A-*b* protein complex that fits the experimental SAXS data best (*χ* = 1.09; Fig. 5b). In this model, the disordered linkers adopt extended conformations. Indeed, in the Kratky-plot of Scaf20L:Cel8A-*b* (Fig. 4b, blue curve), the bell shape maximum is shifted to larger values in comparison to Scaf20L alone (Fig. 4b, green curve), which indicates less globular and more extended regions. Nevertheless, molecular dynamics simulations on this construct revealed that during the simulation, the scaffoldin may also adopt a more compact conformation, which likely represents a minor, transient more-ordered state of the scaffoldin. If present in solution, this form must be very minor, since the experimental solution structure was well-represented by the extended conformer (Fig. 5b).

#### Scaf20L in complex with Cel8A-b, Cel9R-a and Cel48S-t (trivalent DC complex T-DC)

Finally, we studied the complex formed between the Scaf20L scaffoldin and the three divergent, dockerin-bearing enzymes. The *D*_max_ value for the overall complex is 305 ± 15 Å, higher than those of the scaffoldin alone, even if the protein appears to be more globular (Fig. 4b, pink curve). The shape calculations using DAMMIN [44] revealed several different forms, which suggests that the SAXS data cannot be explained by only one conformation.

Since the trivalent T-DC contains several disordered linkers, we expected it to exhibit conformational diversity and flexibility in solution. Therefore, we applied a minimal-ensemble method [46] to the pool of 2 × 10^5^ structural models of the T-DC to gain further structural interpretation of the SAXS data. The minimal ensemble consistent with the SAXS data is a combination of two very distinct models (*χ*^2^ = 1.25; Fig. 5d). One of the models corresponds to an open and elongated conformation of the scaffoldin with a length of 255 Å which is approaching the *D*_max_ determined by SAXS, while the second one represents a compact conformation (155 Å). We can see that the catalytic domains in the two models are mobile. In the first model, *Cel48S-t* and Cel8A-*b* are close to each other, and in the second model, Cel8A-*b* is close to Cel9R*-a*. From the pool of 2 × 10^5^ structural models of the T-DC, the one model that fits best the experimental SAXS data (*χ*^2^ = 1.87; Fig. 5c) shows the disordered linkers in Scaf20L in extended conformations. Although this model does not account for the SAXS data as good as the ensemble of two models (*χ*^2^ = 1.25; Fig. 5d), it was taken as input for MD simulations to further predict the flexibility of the linkers in solution.

#### MD of T-CD

After approximately 30 ns of all-atomistic simulations, the radius of gyration of T-DC is slightly decreased from 7.8 nm to about 7.0 nm. Similar reduction (from 8.6 nm to ~ 7.5 nm) is observed in more coarse-grained simulations, using SIRAH (simulation length: 900 ns) [47–49]. Both the radius of gyration and RMSD of the DC is influenced mainly by the scaffoldin and not by the enzymes (Additional file 7: Fig. S6). The all-atom simulations reveal that the decrease in *R*_g_ is due to a more compact state of the scaffoldin. The individual enzyme structures remain unchanged throughout the simulations, as does the length of the linker between the catalytic domains and their cohesin, indicating that the compaction of the DC is due solely to contraction of the linker into a more compact conformation in the scaffoldin.

### SAXS analyses of the wild-type *C. thermocellum *CipA scaffoldin and its complexation with wild-type *C. thermocellum* Cel8A-*t*

**Fig. 6:**
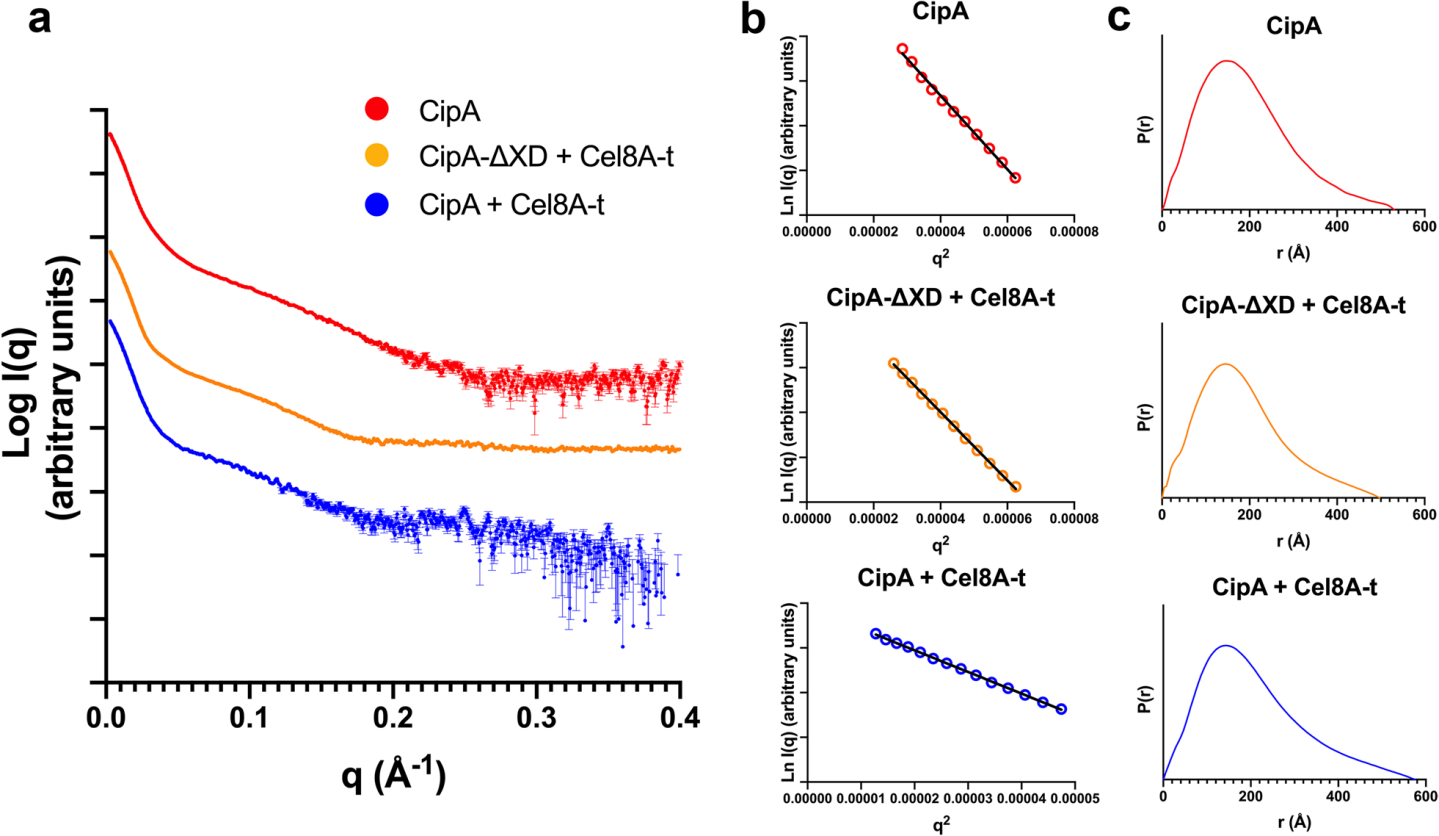
Experimental SAXS data of the *C. thermocellum* CipAs. **a** Experimental scattering curves of CipA alone, CipA-ΔXD in complex with Cel8A-*t*, and finally CipA in complex with Cel8A-*t*; the color codes are given in the legend. **b** Representation of the linear Guinier regions; experimental points are given as open circles (colors as in a) and the black line represents the Guinier-approximation. **c** Representation of the Fourier-transform, P(r)-function, for each of the DC protein and its complexes (colors as in a). See Fig. 1 and Additional file 1: Table S1 for terminology

With the aim of potentially characterizing a cellulosomal complex in a state very close to native, we purified and measured the scattering curves for two *C. thermocellum* CipA constructs, i.e., the full-length CipA (without the signal peptide), both alone and in complex with nine Cel8A-*t* enzymes, and CipA without its X domain (CipA-ΔXD) in complex with nine Cel8A-*t* enzymes (Fig. 6). The scattering curves for CipA-ΔXD alone showed substantial aggregation and clean scattering curves could not be obtained. All samples were collected several times and resulting from different preparations. Figure 6 displays the best and purest scattering curve we could obtain, and *R*_g_ and *D*_max_ values are consistent with the expected solution structure of these macromolecular complexes. They are also consistent with cryo-EM images that were obtained on un-complexed CipA [27, 50]. However, despite several attempts, using various algorithms and strategies to try to model and fit the scattering curves, all efforts remained unsuccessful. We believe that this is due to the large and mostly extended overall form as well as a high flexibility of these complex objects (Additional file 8: Fig. S7). This would produce a potential energy surface littered with a very complex Boltzmann’s population of multiple major and minor conformations, which are not resolvable by the algorithms used to fit and model SAXS curves of mainly compact proteins. Interestingly, and in agreement with the precedent observations on smaller cellulosomal complexes, the *R*_g_ and *D*_max_ values measured for the ‘enzyme-free’ CipA are proportionally larger with respect to the molecular mass than the fully complexed form, indicating more conformations, more flexibility and less compaction for the un-complexed, idle macromolecule (Fig. 6).

## Discussion

Previous studies have shown that dockerin-bearing enzymes in solution are multi-modular objects with substantial flexibility of the linker that separates the dockerin from the other modules, notably the catalytic domain [19, 20, 22, 23]. Significantly, no measurable intermolecular interactions have been revealed in any of the studied cellulosomal enzymes [26]. This is also the case for the solution structure of dockerin-bearing exocellulase Cel48S-*t* and endocellulase Cel8A-*b* in our study (Fig. 2).

Interestingly, the processive endoglucanase Cel9A-*r*, that contains a CBM3c module in addition to the catalytic domain and the dockerin, does not display the same features. As indicated by the smaller *D*_max_ and *R*_g_ than expected (Table 1), this multi-modular enzyme is much more compact and does not appear to reach very extended conformations in solution, in stark contrast to the other two enzymes. Crystal structures of homologous Cel9 enzymes devoid of their dockerins have highlighted that for this type of enzymes the adjacent CBM3c is tightly tethered to the catalytic domain, with essentially no flexibility in their linker [51–54]. Nevertheless, flexibility would be expected for the linker between the CBM and the dockerin. This is not what we observe for the solution structure of Cel9A-*r* (Fig. 2b); here, the linker seems to be pleated against the CBM. It could thus be speculated that the hydrophobic character of the substrate-binding surface of the CBM3c module might be concealed by the linker residues owing to unspecific interactions, such as those observed in ‘fuzzy complexes’ of intrinsically disordered proteins [55–57]. Indeed, pleating of linkers upon increasing the molecular mass of these enzymes has previously been documented for bi-modular enzymes composed of a catalytic domain and a dockerin in complex with their cognate cohesin [20].

Notably, CBM3c—containing GH9 processive cellulases—are recurrent and important enzymes in cellulosomal complexes [45] that might play a key role in further interaction of the overall complex with the insoluble substrate. As such, they are generally present in cellulosomal complexes in higher abundance than other enzymes [58]. In addition, a molecular modeling study involving the self-assembly of the cellulosome enzyme complex [59] has revealed that the binding mechanism of enzymes is dependent on mass and flexibility: larger, multimodular and flexible enzymes (a GH9 homolog in that particular study) exhibit increased binding propensities, compared to smaller quickly diffusing enzymes, thus physically controlling the stoichiometry of integration. Consequently, the more compact form of the Cel9A-*r* observed here might be a minor state, artificially stabilized by the experimental conditions that lead to the pleating of the linker to cover the exposed hydrophobic surface of the CBM3c, and this conformation might be released upon contact with scaffoldins.

Genome mining of cellulosome-producing bacteria has revealed a large variety of cellulosomal systems [60] that potentially are linked to the natural habitats of the micro-organisms [61]. The encountered diversity raises the question whether the composition and spatial organization follows a general rule, or if the diversity also reflects the need to vary the connected biophysical properties, to adapt to specific habitats or substrate sources. In this context, it remains crucial to understand the link between the architecture of cellulosomal systems and their efficiency remains of growing interest. SAXS measurements on several scaffoldins [20, 22, 23, 28, 29], most of them being chimeric constructions, revealed differences in flexible behavior, depending on where the adjacent cohesins are situated within the sequence, with N-terminal cohesins and linkers being more flexible than central ones [28]. In our present study, we expand the SAXS studies of these objects in solution to include three original scaffoldins, which are ScaA, ScaH and ScaK, found in the human gut bacterium *R. champanellensis* [51]. This bacterium is to date the only human colonic bacterium so far reported to efficiently degrade recalcitrant plant polysaccharides, such as crystalline cellulose and xylan [62]. Interestingly, while ScaA can be considered one of the smallest “classical” scaffoldins, consisting of 2 cohesins with an X domain and a dockerin, the other two scaffoldin proteins, ScaH and ScaK, contain catalytic modules within their primary sequences [51]. Since no structural homologues of these modules were available, molecular modeling was not possible for these macromolecules. Nevertheless, *R*_g_ and *D*_max_ values (Table 1), derived from the scattering curves of these proteins in solution, are consistent with rather extended, flexible and multimodular components. Moreover, the Kratky-plots (Fig. 4a) reveal the presence of both extended compact objects, combined with substantial disordered regions. These results are in agreement with the suggestion that these scaffoldins reflect a naturally occurring expansion or diversification of strategies for cohesin–dockerin interactions [63]. These architectural data need now to be completed by single molecule force spectroscopy experiments to demonstrate possible implications of these variations on the complex mechanostability of these interacting proteins [64]. In particular, more work is needed to assess how the balance between compaction and flexibility may be fine-tuned in response to the nature and recalcitrance of the substrate that is targeted and the environment of action. In this context, the presence of unconventional scafoldins, containing peptidases and oxidative enzymes, have been found in *C. alkalicellulosi*, which appear to be associated with both cell-associated and cell-free systems, and might be linked to their occurrence in alkaline soda lake ecosystem [20].

As a next step, the study of artificial designer cellulosomes offers a valuable tool for unraveling synergy-connected architectural features of the complexed cellulosomal enzymes, and may produce to guidelines for design of more efficient and more stable complexes. In the light of the detailed biochemical study of various designer cellulosomes and their efficiency [14] that demonstrated the outstanding performance of Scaf20L in complex with three enzymes, we have explored the overall structural arrangement in solution of this particular cellulosomal complex using the dissect and build strategy with SAXS. Our results on Scaf20L alone, in complex with one single enzyme and in complex with three different enzymes again highlight that ‘loading’ the scaffoldins with enzymes influences the flexibility of the linker regions; the more the complex is loaded, the more compact the overall spatial arrangement becomes (Fig. 4b). The data clearly show that multiple conformers exist in solution, varying between compact forms with pleated linkers and extended conformations, in which the enzymes point away from each other. This spatial arrangement and variability might lay the basis for the mechanics of their plastic action adapted to heterologous catalysis, where the extended conformers are those that stabilize interaction with the (solid) substrate, and the more compact forms maintain the integrity of the complexes in the free and substrate-unbound state, as has been previously proposed [20, 22]. Our findings on the biophysical values of *R*_g_ and *D*_max_ for CipA and its enzyme-complex support this hypothesis. They also confirm the existence of galleries of “loose cellulosome” conformations (Additional file 8: Fig. S7) that have been depicted way back in 1987 by Mayer et al. [27]. The next step would be to further probe the spatial arrangements of these large multi-enzyme complex structures in interaction with a natural, complex substrate, from meso to atomistic scale.

## Conclusions

Understanding the relation between composition and efficiency of cellulosomes, both at the level of sequence and modules, remains a major challenge. Our study underpins the roles of the deformable, mechanically soft architectural arrangements, allowing both compact and extended versions of the macromolecular objects, which are important for the mechanical aspect of their mode of action, and offers a rational basis for engineering more effective next-generation materials. Future work should focus on linking enzymatic synergy on a given complex or natural substrate to these spatial variations, by further examining the catalytic activity and synergy as a function of enzyme position and composition and nature and number of the scaffoldin cohesin–dockerin pairs.

## Methods

### Cloning, protein expression and purification

The cellulosomal scaffoldin and enzyme proteins studied in this work are presented schematically in Fig. 1, and their amino acid sequences are provided in Additional file 1: Table S2. Scaffoldin ScaK scaffoldin from *R. champanellensis* was cloned and purified as previously described by Morais et al*.* [65]. Two additional *R. champanellensis* scaffoldins and selected components, namely, full-length ScaH and its enzymatic SGNH module alone, and full-length ScaA and its X module alone, were cloned using primers listed in Additional file 1: Table S3 and purified using the same protocol [65], except for the X module alone. For this construct, vector pet-28 containing the coding sequence of the X-module was transformed into *E. coli* BL21 (DE3). A pre-culture of the transformed *E. coli* cells in Luria–Bertani (LB) medium was incubated at 37 °C overnight and then diluted at 1:100 in fresh 1L LB medium, containing 5 mM CaCl_2_ for cell growth at 37 °C until reaching an optical density (OD) ~ 0.9. The protein production was induced with 0.2 mM Isopropyl ß-d-1-thiogalactopyran (IPTG) at 16 °C and kept at this temperature for 18 h. Cultures were centrifuged for 35 min at 4 °C, 3000 g. The cell pellet was resuspended in 50 mL of buffer A (TRIS or tris(hydroxymethyl)-aminomethane 30 mM pH 7.5, NaCl 200 mM, 5 mM CaCl_2_) supplemented with 15 µL of DNAse with 6 mM MgSO_4_ and lysed using a French press. Afterwards, the lysate was clarified at 12,000 g for 30 min at 4 °C, and the supernatant was filtered on 0.45 µm. The supernatant was loaded onto a HyperCell PAL column charged with NiCl_2_ (0.1 M) and pre-equilibrated with buffer A that also contained 20 mM imidazole. The column was washed with imidazole containing buffer A. After protein injection, a first step (5 mL) in 140 mM imidazole allowed us to eliminate any unspecific contaminants and denatured fractions, and the protein was then eluted with a linear imidazole gradient produced by the mixing of buffer A and buffer B (TRIS 30 mM pH 7.5, NaCl 150 mM, 5 mM CaCl_2,_ imidazole 1 M) at a flow rate of 1 mL min^−1^. The different fractions were concentrated on an Amicon Ultra 15 (10 kDa) Merck Millipore filter chamber to reach a volume of 2 mL. Finally, the protein was injected onto Sephacryl S-75 size exclusion column (GE Healthcare) pre-equilibrated with buffer C (TRIS 20 mM pH 7.5, NaCl 100 mM, 1 mM CaCl_2_). The protein containing fractions were pooled and concentrated to 30 mg/ml.

The chimeric Scaf20L scaffoldin was cloned and purified as described previously [66–68]. Briefly, the scaffoldin Scaf20L consists of three cohesin domains of divergent specificity and a cellulose-binding module 3a (CBM3a). These include the third cohesin of ScaB from *B. cellulosolvens*, the third cohesin of ScaC from *A. cellulolyticus*, and the second cohesin and CBM3a of the CipA scaffoldin subunit from *C. thermocellum*.

Three cellulases from *C. thermocellum*, containing divergent dockerins to match those of the chimeric scaffoldin, were produced to make the final trivalent designer cellulosome (T-DC). These include the intact, full-length, wild-type Cel48S-*t* enzyme with its own dockerin, Cel9R-*a,* which is the chimeric enzyme containing the fused GH9-CBM3c dyad with a dockerin from *Acetivibrio cellulolyticus* (replacing the wild-type dockerin in the original Cel9R-*t*). Also present is Cel8A-*b,* the chimeric enzyme with a dockerin from *Bacteroides cellulosolvens* (replacing the wild-type dockerin in the original Cel8A-*t*). Cloning, expression and purification of the latter enzymes followed literature procedures [66–68].

Wild-type cellulase Cel9A-*r* from *R. champanellensis* was cloned and purified as described by Morais et al. [36]. Protein production and purification were upscaled to 2 L to produce enough for the SAXS experiments.

The full-length *CipA* gene was synthesized using GenScript® technology on the optimized codon for *E. coli* and was cloned into the pET-51b(+) plasmid between the BamHI and SacI restriction sites. DNA encoding CipA-ΔXD was amplified by PCR using the plasmid encoding the full-length CipA and primers introducing a 5′ SacI restriction site*.* The *Cel8A* gene was amplified by PCR using *C. thermocellum* genomic DNA as template. The gene was subsequently cloned into the pET-21a(+) plasmid between the NheI and XhoI restriction sites. S458 and S459 of the Cel8A dockerin were mutated into alanine using the PCR-based QuikChange method (Stratagene). All the CipA proteins and the Cel8A-t enzyme contain a C-terminal His_6_ tag. To enable the in vivo (*E. coli*) production of the CipA-ΔXD/Cel8A_S458A-S459A_ cellulosomal complex, both genes were expressed from the same plasmid. To do so, the enzyme was first cloned into a pET-3a plasmid using the NdeI and BamHI restriction sites to pick up a T7 promoter and T7 terminator. This was then sub-cloned into pET-51b(+) plasmid also containing the *CipA-*Δ*XD* gene. To do this, the pET-51b(+) plasmid was mutated to add a BglII restriction site upstream of *CipA-*Δ*XD*. Both pET-3a and pET-51b(+) were digested with BglII. The pET51b was subsequently dephosphorylated so that the enzyme insert could then be ligated in. Restriction digest was used to check for correct orientation of the insert. All the primers used are listed in Additional file 1: Table S3. All samples were characterized by dynamic light scattering (DLS) to check monodispersity in solution (data not shown).

### Purification of cellulosomal complexes

The trivalent designer cellulosome (T-DC) is a complex containing stoichiometric concentrations of the chimeric scaffoldin Scaf20L and three *C. thermocellum* cellulases (wild-type Cel48S-*t* and chimeric Cel9R-*a* and Cel8A-*b*, the dockerins of which match the specificities of the three divergent Scaf20L cohesins. The T-DC complex was formed just prior to SAXS analysis, using a molar ratio 1.1:1 of the latter three enzymes relative to the scaffoldin subunit. The complex was then separated from the low levels of residual free components using an SEC-3300 Å column (Agilent Technologies, France).

The full-length, wild-type *C. thermocellum* CipA scaffoldin and the variant without its terminal X-dockerin modular dyad (CipA-ΔXD), both in complex with the wild-type *C. thermocellum* Cel8A-*t* endoglucanase, were purified using an Akta system with a Sephacryl 200 column at the site of the synchrotron facility (Soleil, St Aubin, France), 1 h before injection on the beamline HPLC.

### Small angle X-ray scattering at SWING beamline

The SAXS data were collected at the Synchrotron SOLEIL on the SWING beamline, using an AVIEX170170 CCD detector. Frames were recorded at 12 keV. The sample-to-detector distance was set to 1799 mm for all samples and also to 4000 mm for CipA and its complexes, leading to scattering vectors q ranging from 0.0005 to 0.5 Å^−1^. For all scattering curves, the scattering vector is defined as q = 4π/λ sin θ, where 2θ is the scattering angle. The protein samples were loaded onto a size-exclusion column (Agilent Bio SEC-3 or Bio SEC-5, 4.6 × 300 mm, 3 μm) using the online purification system that delivers the eluted fractions directly into the measurement cell, developed at the SWING beamline [69]. After equilibrating the column with the protein buffer supplemented with 2–5% of radio-protectant (glycerol), 50 µL of protein sample, concentrated at 8 to 15 mg/mL, were injected. Subsequently, and triggered by the elution procedure, a first series of 180 successive frames of 750 ms were recorded on buffer solution (before the column’s void volume) to measure the background. In the next step, 250 frames were collected continuously during the elution, with a frame duration of 1.5 s and a dead time between frames of 0.5 s. In contrast to classical SAXS experiments that are conducted in batch using several protein concentrations within a standard range (e.g., 0.1–10 mg/mL^−1^), here data collection is coupled to a size-exclusion column so that analysis of the required multiple concentrations of the protein occurs within a single experiment. This is because many different positions within the elution peak are sampled during the course of the measurement (typically 50–100 frames are acquired). The averaged buffer scattering curve was then subtracted from the protein signal. *R*_g_ (radius of gyration) values were calculated for each frame during the measurement and those that exhibit the same *R*_g_ were averaged (Additional file 9: Fig. S8). Data reduction to absolute units, frame averaging, and subtraction were performed using the program FOXTROT (Xenocs).

All subsequent data treatment and analysis were performed using Scatter [70] or PRIMUS from the ATSAS suite [71]. The forward scattering *I*(0) and the radius of gyration *R*_g_ were derived by the Guinier approximation *I*(*q*) = *I*(0) exp(− *q*^2^*R*_g_^2/3^) roughly for *qR*_g_ < 1.1 or 1.2 using Scatter. The distance distribution function *P*(*r*) and the maximum particle dimension *D*_max_ were calculated by Fourier inversion of the scattering intensity *I*(*q*) using GNOM [72].

Protein shapes were derived from the experimental SAXS data using the bead-modeling program DAMMIN [44] or GASBOR [73]. At least 20 different calculations were carried out and then aligned with SUPCOMB [74]. The models that had the same shape were averaged using the DAMAVER and DAMFILT packages [71, 75]. The quality of the 3D modeling was determined using the discrepancy *χ*^2^, defined according to Konarev et al. [76]. Values lying in the range of 0.9–1.1 are accepted to indicate a good fit between the models and the data. However, the calculation of *χ*^2^ is inversely proportional to the measurement error. Using the low-error detector at SOLEIL, higher *χ*^2^ values were obtained [77, 78]. Coarse-grain molecular models were then fit into the ab initio envelopes using SUPCOMB [74].

### Coarse-grained molecular modeling of specific components, scaffoldins and complexes

Molecular simulations to study conformations of cellulosomal proteins, in combination with the experimental SAXS curves, were used in a ‘dissect and build’ strategy for four of the studied systems: (1) the full-length wild-type cellulase Cel48S-*t*, (2) a designer scaffoldin Scaf20L, (3) the scaffoldin Scaf20L in complex with the chimeric cellulase Cel8A-*b* and (4) the trivalent designer cellulosome complex (T-DC), consisting of Scaf20L, Cel9R-*a,* Cel8A-*b* and Cel48S-*t*.

To efficiently sample conformations of these four cellulosomal systems, we used coarse-grained (CG) molecular simulations, in which the folded domains of proteins were treated as rigid bodies and the flexible loops and disordered linker segments were modeled by chains of amino-acid beads with appropriate bending, stretching and torsional potentials [79]. To enhance sampling and generate a pool of diverse conformations for SAXS analysis, the replica exchange (RE) method was implemented in Monte Carlo (MC) simulations with replicas at 20 temperatures, ranging from 300 to 500 K. Each of the simulation runs comprised 10^7^ MC sweeps. The simulation structures were saved every 10^3^ MC sweeps. In this way, we generated 2 × 10^5^ structural models for each of the four cellulosomal systems. The scattering intensity profile was computed for each of the structural models individually using the algorithm co-developed with the EROS method [41]. The discrepancy between the experimental SAXS data, *I*_exp_(*q*), and the scattering intensity profile of the *k*th structural model, *I*_*k*_(*q*), was quantified by

$${\chi }_{k}^{2}=\sum_{i=1}^{{N}_{q}}\frac{{(a{I}_{k}\left({q}_{i}\right)+b- {I}_{\mathrm{exp}} ({q}_{i}))}^{2}}{{\sigma }^{2}({q}_{i})}$$

where the index *k* labels the structural models, *N*_*q*_ is the number of SAXS data points, and σ^2^(*q*) is the statistical error of intensity *I*_exp_(*q*). The scale factor *a* and offset *b* result from the conditions ∂*χ*^2^/∂*a* = 0 and ∂*χ*^2^/∂*b* = 0. The offset parameter *b* accounts for uncertainties in the buffer subtraction procedures [80].

### Molecular dynamics

All Molecular Dynamics simulations are carried out with GROMACS 2018 software [81–88]. Two models are considered due to the large size of the system: an all-atom model (CHARMM36m) [89] and a Coarse-Grained (CG) model (SIRAH2) [47–49]. The all-atom model provides detailed insights regarding interactions, in particular hydrogen bonds. The CG model enables long timescale simulations for a more extensive sampling of the DC conformations. All simulations are performed in explicit water and at physiological ionic strength (0.15 M). Additional ions are added to ensure the neutrality of the system. Time steps of 2 fs and 20 fs are used for CHARMM and SIRAH, respectively. Bonds involving a hydrogen atom are constrained with the LINCS algorithm [90]. For both models, the system is first minimized and the heated from 0 to 300 K in the NVT ensemble. Berendsen thermostat [91] is used with a relaxation time of 1 ps. The systems are then equilibrated first in the NVT ensemble and then in the NPT ensemble. Energy fluctuations and evolution of RMSD are shown in Additional file 8: Fig. S7 indicating that the system is well-equilibrated. Production is performed in the NPT ensemble. The V-rescale thermostat [92] and Parrinello–Rahman barostat [93] are used with a relaxation time of 1 ps and 5 ps, respectively. A cutoff of 1.2 nm is used for non-bonding interactions. Electrostatics are computed with the PME scheme.

## Supplementary Information

**Additional file 1: Table S1.** Additional experimental SAXS parameters derived from the scattering curves of the various scaffoldins, components and complexes. **Table S2.** Proteins used in this work and their sequences. Scaffoldin sequences from *R. champanellensis* and *C. thermocellum* are color-coded according to modular content. Modular content of chimaeric scaffoldin and enzymes for preparation of designer cellulosomes is color-coded according to the source species. His represents the position of a His Tag in the specified protein. Molecular weight was calculated using the ProtParam tool (https://web.expasy.org/cgi-bin/protparam/protparam). **Table S3.** Primers and cloning strategy used in this study.

**Additional file 2: Figure S1.** Experimental SAXS data of the individual modules X and SGNH, as well as the enzymes Cel48S-t, Cel8A-b and Cel9A-r. a. Experimental scattering curves; the color codes are given in the legend. b. Representation of the linear Guinier regions; experimental points are given as open circles (colors as in a) and the black line represents the Guinier-approximation c. Representation of the Fourier-transform P(r)-function for each of the modules and enzymes (colors as in a). See Figure 1 and additional Table S1 for terminology.

**Additional file 3: Figure S2.** Left panel: Experimental scattering curve (red points) of Cel9A-*r* from *R. champanellensis* and the best fit obtained by a mixture of the structural models obtained by MD-simulations (black line). Right panel: snapshots of Cel9A-*r *structures obtained by MD-simulations and that best fit the experimental curve with the given proportions (percentage as indicated in the image). The models are represented in blue and the modules composing the protein are indicated as GH9 (catalytic module of Cel9A-r), CBM (CBM3-domain of Cel9A-*r*) and DOC (dockerin of Cel9A-*r*).

**Additional file 4: Figure S3.** GASBOR/DAMMIN-Fit and “solution structure” images of the individual modules X and SGNH a. X-module; experimental curve fitted by GASBOR [73]; b. superimposition of the homology model onto one of the most representative GASBOR envelopes. c. SGNH experimental curve fitted by DAMMIN [43]; d. superimposition of the homology model onto the most representative DAMMIN envelope. e. Kratky plot of the scattering curve of the X-module. f. Kratky plot of the scattering curve of the SGNH module.

**Additional file 5: Figure S4.** Experimental SAXS data of the various wild-type ruminococcal Sca-proteins (ScaA, ScaH and SkaK). a. Experimental scattering curves; the color codes are given in the legend. b. Representation of the linear Guinier regions; experimental points are given as open circles (colors as in a) and the black line represents the Guinier-approximation c. Representation of the Fourier-transform, P(r)-function, for each of the scaffoldin proteins (colors as in a). See Figure 1 and additional Table S1 for terminology.

**Additional file 6: Figure S5.** Experimental SAXS data of the various DCs based on Scaf20L. a. Experimental scattering curves of Scaf20L alone, Scaf20L in complex with Cel8A-*b* and finally Scaf20L in complex with Cel8A-*b*, Cel9R-*a* and Cel48S-*t*; the color codes are given in the legend. b. Representation of the linear Guinier regions; experimental points are given as open circles (colors as in a) and the black line represents the Guinier-approximation c. Representation of the Fourier-transform, P(r)-function, for each of the DC protein and its complexes (colors as in a). See Figure 1 and additional Table S1 for terminology.

**Additional file 7: Figure S6.** Evolution of RMSD (a) DC-complex (Scaf20L in complex with Cel8A-*b*, Cel9R-*a* and Cel48S-*t*) during the time frame of modellization. Energy fluctuations (b) that show that the system is well-equilibrated. (c) Evolution of RMSD of Individual components of the DC-complex (d) Evolution of secondary structure (dssp) during the time frame of modellization (note : DC reaches random coils due to the presence of many long linkers).

**Additional file 8: Figure S7.** Cα-trace representation of a single conformational model of CipA in complex with 9 Cel8A-t enzymes, obtained by coarse-grain molecular modeling.

**Additional file 9: Figure S8.** Experimental SEC–SAXS elution profiles of the major components described in the article, showing I0 vs. Rg values for collected frames. For each data set only images with stable Rg values were averaged to obtain the experimental scattering curve as follows: Cel48S-t image range 160 to 185; Cel8A-b image range 80 to 110; Cel9A-r image range 85 to 105; ScaA image range 2 to 80; ScaH image range 50 to 95; Scaf20L alone image range 65 to 100; Scaf20L in complex with Cel8A, Cel9R and Cel48S image range 150 to 210; CipA image range 180 to 210; CipA+Cel8A-t image range 75 to 115; CipA-ΔXD+Cel8A-t image range 150 to 175.

## Data Availability

All the supporting data are available.
