# Supplementary material for: Mapping the deformability of natural and designed cellulosomes in solution
Source: Biotechnol Biofuels Bioprod. 2022 Jun 20;15:68. doi: 10.1186/s13068-022-02165-3 (PMC9210761; doi:10.1186/s13068-022-02165-3)
Supplement: Supplementary file 1 — Additional file 1: Table S1. Additional experimental SAXS parameters derived from the scattering curves of the various scaffoldins, components and complexes. Table S2. Proteins used in this work and their sequences. Scaffoldin sequences from R. champanellensis and C. thermocellum are color-coded according to modular content. Modular content of chimaeric scaffoldin and enzymes for preparation of designer cellulosomes is color-coded according to the source species. His represents the position of a His Tag in the specified protein. Molecular weight was calculated using the ProtParam tool (https://web.expasy.org/cgi-bin/protparam/protparam). Table S3. Primers and cloning strategy used in this study. [file 13068_2022_2165_MOESM1_ESM.docx]

**Supplemental Information**

**Mapping the deformability of natural and designed cellulosomes in solution**

Jonathan Dorival^1,2^, Sarah Moraïs^3,4^, Aurore Labourel^5^, Bartosz Rozycki^6^, Pierre A Cazade^7^, Jérôme Dabin^1^, Eva Setter-Lamed^3^, Itzhak Mizrahi^4^, Damien Thompson^7^, Aurélien Thureau^8^, Edward A Bayer^3,4^, Mirjam Czjzek^1^

Affiliations

^1^Sorbonne Université, CNRS, Integrative Biology of Marine Models (LBI2M), Station Biologique de Roscoff (SBR), 29680 Roscoff, Bretagne, France

^2^Department of Biological Sciences, Vanderbilt University, Nashville, TN 37232, USA

^3^Department of Biomolecular Sciences, The Weizmann Institute of Science, 7610001 Rehovot, Israel

^4^Faculty of Natural Sciences, Ben-Gurion University of the Negev, Beer-Sheva 8499000 Israel

^5^TBI, Université de Toulouse, CNRS, INRAE, INSA, Toulouse, France

^6^Institute of Physics, Polish Academy of Sciences, Al. Lotnikow 32/46, 02-668 Warsaw, Poland

^7^ Department of Physics, Bernal Institute, University of Limerick, Limerick, Ireland.

**^8^**Synchrotron SOLEIL, 91190 Saint Aubin, France

**Additional Results**

**1. SAXS analyses of individual modules, enzymes and components**

*X-module, SGNH module*

We have also collected SAXS data for those modules for which no crystal structures were available, namely the X module of *R. champanellensis* ScaA and the SGNH module present in ScaH (additional Figure S3 and Table S1). As expected, the scattering curves (additional Figure S3a and S3c) and Kratky-plots (additional Figure S3e and S3f) of both of these modules are indicative of compact objects in solution, with an R_g_ of 22.6 and 27.1 Å, respectively.

Despite multiple attempts we were not able to crystallize the X-module, instead we used recently developed structure prediction to create a 3D model of this module with Robetta (http://robetta.bakerlab.org/), which displays a shape that fits the envelope obtained from SAXS data we acquired on the X module alone. The SAXS envelope of the isolated X-module confirms that it is a compact protein with an oval or diamond shape (additional Figure S3b).

**2. SAXS measurements of scaffoldin variants**

In order to acquire more detail on the multimodular ScaH protein, we first acquired SAXS data on the isolated SGNH domain (additional Figure S3c). The overall shape calculated with DAMMIN for this protein was surprising, showing a globular domain but also an extended tail (additional Figure S3d). To further understand this phenomenon, we have generated homology models. As SGNH has homology with esterases and lipases, we have generated two different models based on the PDB structures: 1ESE (esterase)[1] and 3BZW (lipase). Only the model based on the structure of the lipase fits the SAXS data (χ^2^=3.35 compared to χ^2^=7.40 for the model based on the esterase). The superimposition of the lipase-based model onto the SAXS shape of SGNH alone is displayed in additional Figure S3d. The main difference between these two model proteins is the presence of a long β-hairpin in the lipase, which fits in the tail of the global shape calculated by SAXS (additional Figure S3d). However, it is difficult to define the position of the SGNH domain in the SAXS envelope of ScaH, probably because of the intrinsic partial flexibility of the entire protein in solution that leads to a loss of resolution.

[1]. Wei Y, Schottel JL, Derewenda U, Swenson L, Patkar S, Derewenda ZS. A novel variant of the catalytic triad in the Streptomyces scabies esterase. Nat Struct Mol Biol. 1995;2(3):218–23.

**Additional Figures**

Additional Figure S1. Experimental SAXS data of the individual modules X and SGNH, as well as the enzymes Cel48S-t, Cel8A-b and Cel9A-r. **a**. Experimental scattering curves; the color codes are given in the legend. **b**. Representation of the linear Guinier regions; experimental points are given as open circles (colors as in **a**) and the black line represents the Guinier-approximation **c.** Representation of the Fourier-transform P(r)-function for each of the modules and enzymes (colors as in **a**). See Figure 1 and additional Table S1 for terminology.

Additional Figure S2. Left panel: Experimental scattering curve (red points) of Cel9A-*r* from *R. champanellensis* and the best fit obtained by a mixture of the structural models obtained by MD-simulations (black line). Right panel: snapshots of Cel9A-*r* structures obtained by MD-simulations and that best fit the experimental curve with the given proportions (percentage as indicated in the image). The models are represented in blue and the modules composing the protein are indicated as GH9 (catalytic module of Cel9A-r), CBM (CBM3-domain of Cel9A-*r*) and DOC (dockerin of Cel9A-*r*).

Additional Figure S3. GASBOR/DAMMIN-Fit and “solution structure” images of the individual modules X and SGNH **a**. X-module; experimental curve fitted by GASBOR [73]; **b.** superimposition of the homology model onto one of the most representative GASBOR envelopes. **c**. SGNH experimental curve fitted by DAMMIN [43]; **d.** superimposition of the homology model onto the most representative DAMMIN envelope. **e.** Kratky plot of the scattering curve of the X-module. **f**. Kratky plot of the scattering curve of the SGNH module.

Additional Figure S4. Experimental SAXS data of the various wild-type ruminococcal Sca-proteins (ScaA, ScaH and SkaK). **a**. Experimental scattering curves; the color codes are given in the legend. **b**. Representation of the linear Guinier regions; experimental points are given as open circles (colors as in **a**) and the black line represents the Guinier-approximation **c.** Representation of the Fourier-transform, P(r)-function, for each of the scaffoldin proteins (colors as in **a**). See Figure 1 and additional Table S1 for terminology.

Additional Figure S5. Experimental SAXS data of the various DCs based on Scaf20L. **a**. Experimental scattering curves of Scaf20L alone, Scaf20L in complex with Cel8A-*b* and finally Scaf20L in complex with Cel8A-*b*, Cel9R-*a* and Cel48S-*t*; the color codes are given in the legend. **b**. Representation of the linear Guinier regions; experimental points are given as open circles (colors as in **a**) and the black line represents the Guinier-approximation **c.** Representation of the Fourier-transform, P(r)-function, for each of the DC protein and its complexes (colors as in **a**). See Figure 1 and additional Table S1 for terminology.

Additional Figure S6. Evolution of RMSD (**a**) DC-complex (Scaf20L in complex with Cel8A-*b*, Cel9R-*a* and Cel48S-*t*) during the time frame of modellization. Energy fluctuations (**b**) that show that the system is well-equilibrated. (**c**) Evolution of RMSD of Individual components of the DC-complex (**d**) Evolution of secondary structure (dssp) during the time frame of modellization (note : DC reaches random coils due to the presence of many long linkers).

Additional Figure S7. Cα-trace representation of a single conformational model of CipA in complex with 9 Cel8A-t enzymes, obtained by coarse-grain molecular modeling.

Additional Figure S8. Experimental SEC-SAXS elution profiles of the major components described in the article, showing I0 versus Rg values for collected frames. For each data set only images with stable Rg values were averaged to obtain the experimental scattering curve as follows : **Cel48S-t** image range 160 to 185; **Cel8A-b** image range 80 to 110; **Cel9A-r** image range 85 to 105; **ScaA** image range 2 to 80; **ScaH** image range 50 to 95; **Scaf20L alone** image range 65 to 100; **Scaf20L in complex with Cel8A, Cel9R and Cel48S** image range 150 to 210; **CipA** image range 180 to 210; **CipA+Cel8A-t** image range 75 to 115; **CipA-ΔXD+Cel8A-t** image range 150 to 175.

**Additional Tables**

**Table S1. Additional experimental SAXS parameters derived from the scattering curves of the various scaffoldins, components and complexes**

| **Construct** | **organism** | **R_g_**  (Å) | **D_max_**  (Å) | **M_w_**  (kDa) | **q**R_g_ | **Porod Volume**  **(**e^+3^) | **I(0)** | R_g_ **from P(r)**  (Å) | **χ^2^ of fit** | **Crysol fit** |
| --- | --- | --- | --- | --- | --- | --- | --- | --- | --- | --- |
| **Scaffoldins & components** | |  |  |  |  |  |  |  |  |  |
| ScaA | *R. champanellensis* | 60.4 ± 0.2 | 282 ± 11 | 68.7 | 1.0 | 166.5 | 0.116 | 65.9 | 3.1 | - |
| X module of ScaA | *R. champanellensis* | 21.6 ± 0.1 | 91 ± 5 | 23.2 | 0.96 | 63.9 | 27.3 | 22.2 | 3.7 | 21.1 |
| ScaH | *R. champanellensis* | 55.8 ± 0.2 | 230 ± 15 | 55.1 | 1.13 | 171.6 | 0.0206 | 59.7 | 3.1 | - |
| SGNH module of ScaH | *R. champanellensis* | 27.1 ± 0.6 | 103 ± 5 | 29.3 | 0.88 | 74.5 | 0.014 | 27.2 | 2.6 | 3.35 |
| ScaK | *R. champanellensis* | 44.9 ± 0.2 | 184 ± 6 | 52.3 | 1.1 | 98.5 | 14.8 | 46.6 | 5.3 | - |
| Scaf20L | Chimeric scaffoldin: CBM and cohesin from *C. thermocellum,* cohesins from *A. cellulolyticus* and *B. cellulosolvens* | 66.3 ± 0.4 | 262 ± 10 | 75.3 | 1.12 | 203.4 | 0.037 | 70.4 | 1.1 | - |
| **Enzymes** |  |  |  |  |  |  |  |  |  |  |
| Cel48S-*t* | Wild-type *GH48S* and *dockerin* from *C. thermocellum* | 34.2 ± 0.1 | 148 ± 4 | 81.6 | 0.92 | 123.9 | 0.027 | 34.9 | 1.3 | 2.2 |
| Cel8A-*b* | Chimeric enzyme: GH8 from *C. thermocellum,* dockerin from *B. cellulosolvens* | 29.9 ± 0.3 | 118 ± 3 | 51.6 | 0.93 | 78.8 | 0.002 | 29.9 | 8.3 | 6.9 |
| Cel9A-*r* | Wild-type enzyme, GH9-CBM3c and dockerin from *R. champanellensis* | 35.2 ± 0.1 | 110 ± 3 | 91.8 | 1.12 | 120.7 | 0.0082 | 35.2 | 5.5 | 4.1 |
| **DC Complexes** |  |  |  |  |  |  |  |  |  |  |
| Scaf20L + Cel8A-*b* | Chimeric scaffoldin and chimaeric enzyme | 64.3 ± 0.3 | 251 ± 8 | 126.9 | 1.17 | 213.6 | 0.053 | 67.5 | 1.1 | - |
| Scaf20L + Cel9R-*a* + Cel8A-*b* + Cel48S-*t* | Chimeric scaffoldin and chimaeric and wild-type enzymes from *C. thermocellum* bearing dockerins that match the cohesins of Scaf20L | 90.9 ± 0.5 | 305 ± 15 | 300.3 | 1.08 | 501.7 | 0.082 | 90.3 | 1.3 | - |
| **CipA & complex** | |  |  |  |  |  |  |  |  |  |
| CipA | Wild-type scaffoldin from *C. thermocellum* | 157 ± 1.6 | 530 ± 20 | 198.1 | 1.24 | 2649 | 0.392 | 154.9 | ND | ND |
| CipA-ΔXD + Cel8A-*t* | Truncated scaffoldin and wild-type enzyme from *C. thermocellum* | 151 ± 1.7 | 497 ± 18 | 632.2 | 1.19 | 674.5 | 0.0001 | 140.7 | ND | ND |
| CipA + Cel8A-*t* | Wild-type scaffoldin and wild-type enzyme from *C. thermocellum* | 170.1 ± 1.2 | 575 ± 20 | 651.7 | 1.09 | 1364 | 0.0004 | 168.0 | ND | ND |

**Table S2. Proteins used in this work and their sequences.** Scaffoldin sequences from *R. champanellensis* and *C. thermocellum* are color-coded according to modular content. Modular content of chimaeric scaffoldin and enzymes for preparation of designer cellulosomes is color-coded according to the source species. His represents the position of a His Tag in the specified protein. Molecular weight was calculated using the ProtParam tool (https://web.expasy.org/cgi-bin/protparam/protparam).

| **Protein** | **Origin** | **Modular content** | **Mol. Weight**  **(Daltons)** | **Amino acid sequence** |
| --- | --- | --- | --- | --- |
| ScaA | *R. champanellensis* | **His-X-Coh-Coh-Doc** | 68,728 | MGHHHHHHAGELEFTFKERSTSSTTFNISAADLAAGDVTVDCDVIITNYVPTYGFGLKMDFENTDTNTIYDASTAENPYFAFTEKTQFDKTGPFTDVWGVEDGTYEPQPGLSATKIANYSMMNFVWRTAATLDDDSTPTNAYFIPDTTNPLLNISFTVASDTPAGTYILDIKTEPYTNTTGGAVSTGQSKINNVDGEVVPFKTVPLTIVVGDAATTTTTAAATTTKAPATTTTAAPATTTKAPTTTTKSEAPVGGVVYEIATVEGEAGADVDVPITIKGDTGTAGMVLEMSADSNLKLKRRLNGDAYEGAPTWNKDTLTYVWNAGDGRNLVAADGAVLTTLKFTVPADAQPGDEYPISFRSDLCKVIDQEGVELNITYVDGMIKIPGEATTTTAAPATTTKAPTTTTKSEAPVGGVVYEIATVEGEAGADVDVPITIKGDTGTAGMVLEMSADSNLKLKRRLNGDAYEGAPTWNKDTLTYVWNAGDGRNLVAADGAVLTTLKFTVPADAQPGDEYPISFRSDLCKVIDQEGVELNITYVDGKIVIPGTATTTKAPATTTSEAPATTTSTQTTTTAPVGTVLYGDTNVDGRVSIADAVLLNKYLAGKAEMTEQGKINADCDKHSTELNLDDTTMILKFLAQLIEQSDLGAEVA |
| X module of ScaA | *R. champanellensis* | **His-X** | 23,224 | MGHHHHHHAGELEFTFKERSTSSTTFNISAADLAAGDVTVDCDVIITNYVPTYGFGLKMDFENTDTNTIYDASTAENPYFAFTEKTQFDKTGPFTDVWGVEDGTYEPQPGLSATKIANYSMMNFVWRTAATLDDDSTPTNAYFIPDTTNPLLNISFTVASDTPAGTYILDIKTEPYTNTTGGAVSTGQSKINNVDGEVVPFKTVPLTIVVGDAA |
| ScaH | *R. champanellensis* | **His-SGNH-Coh-Doc** | 55,074 | MAHHHHHHEQDPKYILMLGDSIASGYGLAEGEYRYADYLEEYLGMESIDYAKPGQTTGELLELVNNEEVQIDIPLASVICVSIGGNDLIDTVEGYLNTLLETYNTTNGTSLTLKEYVQTVVAVDDDLQTTMILKLTSLLNKAANTYKTNIQQIEASLLEQNPDAKIVVQTVYNPINMENPVVNGVDYSSKLKQIRKFASEQLLTLNDALQQTEGLTYVDVNAAFKDTEWVYTNMDPSNGFWQMDVHPNALGHAVIAAEILNSLGAEGGSCDQFNLVLLNNAAKLSDAEYDRVHAQLDNFITTGKDGIVYSIAGVKGKPGETVDVPITISGDTGTAGMVLELQADAGLTIKRRVTGNAYEGAPTWNPKTLTYVWNTADGRNQVAADGAVLATLQFTIAEDAVNGMYEISFDEAKCDIVDENGTALDVTFENGGVEVYGSTVPEYKLGDVNMDGALTVADAVTVLQACAQVTAGGESPLTDQQKKLADMNQDGNVSVGDAVDILVTIAQSMVG |
| SGNH module of ScaH | *R. champanellensis* | **His-SGNH** | 29,362 | MTHHHHHHEQDPKYILMLGDSIASGYGLAEGEYRYADYLEEYLGMESIDYAKPGQTTGELLELVNNEEVQIDIPLASVICVSIGGNDLIDTVEGYLNTLLETYNTTNGTSLTLKEYVQTVVAVDDDLQTTMILKLTSLLNKAANTYKTNIQQIEASLLEQNPDAKIVVQTVYNPINMENPVVNGVDYSSKLKQIRKFASEQLLTLNDALQQTEGLTYVDVNAAFKDTEWVYTNMDPSNGFWQMDVHPNALGHAVIAAEILNSLG |
| ScaK | *R. champanellensis* | **His-Coh-GH25** | 52,326 | MAHHHHHHADQTVQTGDIAVPEVSVGTDAVAEQTSDVLNVSTKDINDTEGDLTETIRLPSCAQINQAICKSTQEPAADDTYDIDGNGVINAFDNVLRKRQLLESQSEYAHLFVSKAVGYGGDVVPVTVSVSGNPGFQNFIMSFSMTQGDYLTMQTGEDGNLKLTQPDQDLTLRAVSGGNVAAVYSTSAVRYTDNGELFTVYVEIPEDTPVGVYPLEMVVQSIEESGNQKVPYVITQGTVTVREEVILPPVTTTTTTVPLETTTTTTTTTTSTGPKGQIYDGIDVSKWQGTVDWAKVKADGYHFAIIRAGYGREASQVDPTFATNVAGAKKAGLYCGAYWYSYATDAAGAKAEAELFLKTVKGYQFDFPLVFDIEDSTQQSLSKSTVAAIIDTFCSTVENAGYYCTLYSYASFLTNNVPVSCQSEHDIWVAHTKTEKPAFSRAYGMWQYSHTGTVNGVSGSTDLNYAYKDYPAIMQKYGFNGF |
| Scaf20L | Chimeric scaffoldin: CBM and cohesin from *C. thermocellum,* cohesins from *A. cellulolyticus* and *B. cellulosolvens* | **CBM-CohA-CohB-CohT-His** | 74,693 | MANTPVSGNLKVEFYNSNPSDTTNSINPQFKVTNTGSSAIDLSKLTLRYYYTVDGQKDQTFWCDHAAIIGSNGSYNGITSNVKGTFVKMSSSTNNADTYLEISFTGGTLEPGAHVQIQGRFAKNDWSNYTQSNDYSFKSASQFVEWDQVTAYLNGVLVWGKEPGGSVVPSTQPVTTPPATTKPPATTKPPATTIPPSGSDLQVDIGSTSGKAGSVVSVPITFTNVPKSGIYALSFRTNFDPQKVTVASIDAGSLIENASDFTTYYNNENGFASMTFEAPVDRARIIDSDGVFATINFKVSDSAKVGELYNITTNSAYTSFYYSGTDEIKNVVYNDGKIEVIASPTPTQSATPTVTPSATATPTQSATPTVTPSSPGNKMKIQIGDVKANQGDTVIVPITFNEVPVMGVNNCNFTLAYDKNIMEFISADAGDIVTLPMANYSYNMPSDGLVKFLYNDQAQGAMSIKEDGTFANVKFKIKQSAAFGKYSVGIKAIGSISALSNSKLIPIESIFKDGSITVTNTPTNTISVTPTNNSTPTNNSTPKPNPLSDGVVVEIGKVTGSVGTTVEIPVYFRGVPSKGIANCDFVFRYDPNVLEIIGIDPGDIIVDPNPTKSFDTAIYPDRKIIVFLFAEDSGTGAYAITKDGVFAKIRATVKSSAPGYITFDEVGGFADNDLVEQKVSFIDGGVNVGNATLEHHHHHH |
| Cel8A-*t* | Wild-type Cel8A from *C. thermocellum* | **GH8-Doc*t*-His** | 50,337 | MGVPFNTKYPYGPTSIADNQSEVTAMLKAEWEDWKSKRITSNGAGGYKRVQRDASTNYDTVSEGMGYGLLLAVCFNEQALFDDLYRYVKSHFNGNGLMHWHIDANNNVTSHDGGDGAATDADEDIALALIFADKLWGSSGAINYGQEARTLINNLYNHCVEHGSYVLKPGDRWGGSSVTNPSYFAPAWYKVYAQYTGDTRWNQVADKCYQIVEEVKKYNNGTGLVPDWCTASGTPASGQSYDYKYDATRYGWRTAVDYSWFGDQRAKANCDMLTKFFARDGAKGIVDGYTIQGSKISNNHNASFIGPVAAASMTGYDLNFAKELYRETVAVKDSEYYGYYGNSLRLLTLLYITGNFPNPLSDLSGQPTPPSNPTPSLPPQVVYGDVNGDGNVNSTDLTMLKRYLLKSVTNINREAADVNRDGAINSSDMTILKRYLIKSIPHLPYLEHHHHHH |
| Cel8A-*b* | Chimeric enzyme: GH8 from *C. thermocellum,* dockerin from *B. cellulosolvens* | **GH8-Doc*b*-His** | 51,669 | MGVPFNTKYPYGPTSIADNQSEVTAMLKAEWEDWKSKRITSNGAGGYKRVQRDASTNYDTVSEGMGYGLLLAVCFNEQALFDDLYRYVKSHFNGNGLMHWHIDANNNVTSHDGGDGAATDADEDIALALIFADKLWGSSGAINYGQEARTLINNLYNHCVEHGSYVLKPGDRWGGSSVTNPSYFAPAWYKVYAQYTGDTRWNQVADKCYQIVEEVKKYNNGTGLVPDWCTASGTPASGQSYDYKYDATRYGWRTAVDYSWFGDQRAKANCDMLTKFFARDGAKGIVDGYTIQGSKISNNHNASFIGPVAAASMTGYDLNFAKELYRETVAVKDSEYYGYYGNSLRLLTLLYITGNFPNPLSDLSGQPTPPSNPTPSLVPPKGTATVLYGDVDNDGNVDSDDYAYMRQWLIGMIADFPGGDIGLANADVDGDGNVDSDDYAYMRQWLIGMISEFPAEQKALEHHHHHH |
| Cel9A-*r* | Wild-type Cel9A from *R. champanellensis* | **His-GH9-CBM3c-Doc*r*** | 91,825 | MAHHHHHHLCLPGAAPQGGDLLANAAGDAASGFDANFAKLLQYSIYFYDANMCGTDVSENNRLNWRGDCHTYDAQVPMDTEHTNLSSAFLTANKDYLDPDGDGFIDVSGGFHDAGDHVKFGMPENYSAATVGWGYYEFRDAYAATGQDAHVETILRYFNDYLMRCTFLDDSGDVVAFCYQVGDGDIDHAYWQAPEIDTMDRPAFFLTGDKPQTDYVASAAASLAINYLNFKDTDEAYAAKSLKYANALYDFARDHEKELSDNGDGPKQYYSSSKWQDDYCWASAWMYKITGDHAYLEEIYPNYDYYAAPCYVYCWNDMWGGVQCVLGEIVSEMYPNFIDEYKEAAGKSPYEEMDCWASVKEALDTYMSGGIGEISPQGYFWLNTWGSARYNTAAQLIAMVYDKYTNNNQPSKYSDWAKGQMEYLMGNNDITYQERIDANTEAENSGNPAPYSADELHGPRCFIVGFNDVAAAYPHHRASSGLSKCEDTKPQKHVLVGALVGGPDNKDLHNDVTKDWIYNEVTIDYNAAFVGASAGLYHFYGTDAMQPDPDIDLGTSEEEGGGQDYWVEAYAVDDKQTSGAGVTKLAMLVCTDSNKPRTDISVRYYFSVKELSNPSNVSLVKGDELYDQTSVETDFDGVLSGPYQYDASFDPDIYYIEVKWDGYNIANANKKYQLAVGFYYGDTWDPTNDWSYQGITKCKDTYQDGSETRTDYICVYSGDTLVGGIEPNGSKPVVTTAATTEGSGTTTTTTTTTTDTTVLGDVDGNGKVEVNDLVRLARYVAQDQELTPALTAQQVTNADVNCDGTVDASDITMIARALARLTSLEDFGK |
| Cel9R-*a* | Chimaeric enzyme, GH9-CBM3c from Cel9R of *C. thermocellum* and dockerin from *A. cellulolyticus* | **GH9-CBM3c-Doc*a*-His** | 78,711 | MADYNYGEALQKAIMFYEFQMSGKLPDNIRNNWRGDSCLGDGSDVGLDLTGGWFDAGDHVKFNLPMAYTATMLAWAVYEYKDALQKSGQLGYLMDQIKWASDYFIRCHPEKYVYYYQVGNGDMDHRWWVPAECIDVQAPRPSYKVDLSNPGSTVTAGTAAALAATALVFKDTDPAYAALCIRHAKELFDFAETTMSDKGYTAALNFYTSHSGWYDELSWAGAWIYLADGDETYLEKAEKYVDKWPIESQTTYIAYSWGHCWDDVHYGAALLLAKITNKSLYKEAIERHLDYWTVGFNGQRVRYTPKGLAHLTDWGVLRHATTTAFLACVYSDWSECPREKANIYIDFAKKQADYALGSSGRSYVVGFGVNPPQHPHHRTAHSSWCDSQKVPEYHRHVLYGALVGGPDASDAYVDDIGNYVTNEVACDYNAGFVGLLAKMYEKYGGNPIPNFMAIEEKTNEEIYVEATANSNNGVELKTYLYNKSGWPARVCDKLSFRYFMDLTEYVSAGYNPNDITVSIIYSAAPTAKISKPILYDASKNIYYCEIDLSGTKIFPGSNSDHQKETQFRIQPPAGAPWDNTNDFSYQGIKKNGEVVKEMPVYEDGVLIFGVEPNGTDPKFIYGDVDGNGSVRINDAVLIRDYVLGKINEFPYEYGMLAADVDGNGSIKINDAVLVRDYVLGKIFLFPVEEKELEHHHHHH |
| Cel48S-*t* | Wild-type Cel48S from *C. thermocellum* | **GH48-Doc*t*-His** | 81,625 | MGPTKAPTKDGTSYKDLFLELYGKIKDPKNGYFSPDEGIPYHSIETLIVEAPDYGHVTTSEAFSYYVWLEAMYGNLTGNWSGVETAWKVMEDWIIPDSTEQPGMSSYNPNSPATYADEYEDPSYYPSELKFDTVRVGSDPVHNDLVSAYGPNMYLMHWLMDVDNWYGFGTGTRATFINTFQRGEQESTWETIPHPSIEEFKYGGPNGFLDLFTKDRSYAKQWRYTNAPDAEGRAIQAVYWANKWAKEQGKGSAVASVVSKAAKMGDFLRNDMFDKYFMKIGAQDKTPATGYDSAHYLMAWYTAWGGGIGASWAWKIGCSHAHFGYQNPFQGWVSATQSDFAPKSSNGKRDWTTSYKRQLEFYQWLQSAEGGIAGGATNSWNGRYEKYPAGTSTFYGMAYVPHPVYADPGSNQWFGFQAWSMQRVMEYYLETGDSSVKNLIKKWVDWVMSEIKLYDDGTFAIPSDLEWSGQPDTWTGTYTGNPNLHVRVTSYGTDLGVAGSLANALATYAAATERWEGKLDTKARDMAAELVNRAWYNFYCSEGKGVVTEEARADYKRFFEQEVYVPAGWSGTMPNGDKIQPGIKFIDIRTKYRQDPYYDIVYQAYLRGEAPVLNYHRFWHEVDLAVAMGVLATYFPDMTYKVPGTPSTKLYGDVNDDGKVNSTDAVALKRYVLRSGISINTDNADLNEDGRVNSTDLGILKRYILKEIDTLPYKNHHHHHH |
| CipA | Full-length, wild-type scaffoldin from *C. thermocellum* | **Coh-Coh-CBM-Coh-Coh-Coh-Coh-Coh-Coh-Coh-XDoc-His** | 198,143 | MASWSHPQFEKGADDDDKVPDATMTVEIGKVTAAVGSKVEIPITLKGVPSKGMANCDFVLGYDPNVLEVTEVKPGSIIKDPDPSKSFDSAIYPDRKMIVFLFAEDSGRGTYAITQDGVFATIVATVKSAAAAPITLLEVGAFADNDLVEISTTFVAGGVNLGSSVPTTQPNVPSDGVVVEIGKVTGSVGTTVEIPVYFRGVPSKGIANCDFVFRYDPNVLEIIGIDPGDIIVDPNPTKSFDTAIYPDRKIIVFLFAEDSGTGAYAITKDGVFAKIRATVKSSAPGYITFDEVGGFADNDLVEQKVSFIDGGVNVGNATPTKGATPTNTATPTKSATATPTRPSVPTNTPTNTPANTPVSGNLKVEFYNSNPSDTTNSINPQFKVTNTGSSAIDLSKLTLRYYYTVDGQKDQTFWCDHAAIIGSNGSYNGITSNVKGTFVKMSSSTNNADTYLEISFTGGTLEPGAHVQIQGRFAKNDWSNYTQSNDYSFKSASQFVEWDQVTAYLNGVLVWGKEPGGSVVPSTQPVTTPPATTKPPATTKPPATTIPPSDDPNAIKIKVDTVNAKPGDTVNIPVRFSGIPSKGIANCDFVYSYDPNVLEIIEIKPGELIVDPNPDKSFDTAVYPDRKIIVFLFAEDSGTGAYAITKDGVFATIVAKVKSGAPNGLSVIKFVEVGGFANNDLVEQRTQFFDGGVNVGDTTVPTTPTTPVTTPTDDSNAVRIKVDTVNAKPGDTVRIPVRFSGIPSKGIANCDFVYSYDPNVLEIIEIEPGDIIVDPNPDKSFDTAVYPDRKIIVFLFAEDSGTGAYAITKDGVFATIVAKVKSGAPNGLSVIKFVEVGGFANNDLVEQKTQFFDGGVNVGDTTEPATPTTPVTTPTTTDDLDAVRIKVDTVNAKPGDTVRIPVRFSGIPSKGIANCDFVYSYDPNVLEIIEIEPGDIIVDPNPDKSFDTAVYPDRKIIVFLFAEDSGTGAYAITKDGVFATIVAKVKSGAPNGLSVIKFVEVGGFANNDLVEQKTQFFDGGVNVGDTTEPATPTTPVTTPTTTDDLDAVRIKVDTVNAKPGDTVRIPVRFSGIPSKGIANCDFVYSYDPNVLEIIEIEPGDIIVDPNPDKSFDTAVYPDRKIIVFLFAEDSGTGAYAITKDGVFATIVAKVKEGAPNGLSVIKFVEVGGFANNDLVEQKTQFFDGGVNVGDTTEPATPTTPVTTPTTTDDLDAVRIKVDTVNAKPGDTVRIPVRFSGIPSKGIANCDFVYSYDPNVLEIIEIEPGELIVDPNPTKSFDTAVYPDRKMIVFLFAEDSGTGAYAITEDGVFATIVAKVKSGAPNGLSVIKFVEVGGFANNDLVEQKTQFFDGGVNVGDTTEPATPTTPVTTPTTTDDLDAVRIKVDTVNAKPGDTVRIPVRFSGIPSKGIANCDFVYSYDPNVLEIIEIEPGDIIVDPNPDKSFDTAVYPDRKIIVFLFAEDSGTGAYAITKDGVFATIVAKVKEGAPNGLSVIKFVEVGGFANNDLVEQKTQFFDGGVNVGDTTVPTTSPTTTPPEPTITPNKLTLKIGRAEGRPGDTVEIPVNLYGVPQKGIASGDFVVSYDPNVLEIIEIEPGELIVDPNPTKSFDTAVYPDRKMIVFLFAEDSGTGAYAITEDGVFATIVAKVKEGAPEGFSAIEISEFGAFADNDLVEVETDLINGGVLVTNKPVIEGYKVSGYILPDFSFDATVAPLVKAGFKVEIVGTELYAVTDANGYFEITGVPANASGYTLKISRATYLDRVIANVVVTGDTSVSTSQAPIMMWVGDIVKDNSINLLDVAEVIRCFNATKGSANYVEELDINRNGAINMQDIMIVHKHFGATSSDYDAQGAPGFSSISAHHHHHHHH |
| CipA-ΔXD | Truncated scaffoldin from *C. thermocellum* | **Coh-Coh-CBM-Coh-Coh-Coh-Coh-Coh-Coh-Coh-His** | 180,440 | MASWSHPQFEKGADDDDKVPDATMTVEIGKVTAAVGSKVEIPITLKGVPSKGMANCDFVLGYDPNVLEVTEVKPGSIIKDPDPSKSFDSAIYPDRKMIVFLFAEDSGRGTYAITQDGVFATIVATVKSAAAAPITLLEVGAFADNDLVEISTTFVAGGVNLGSSVPTTQPNVPSDGVVVEIGKVTGSVGTTVEIPVYFRGVPSKGIANCDFVFRYDPNVLEIIGIDPGDIIVDPNPTKSFDTAIYPDRKIIVFLFAEDSGTGAYAITKDGVFAKIRATVKSSAPGYITFDEVGGFADNDLVEQKVSFIDGGVNVGNATPTKGATPTNTATPTKSATATPTRPSVPTNTPTNTPANTPVSGNLKVEFYNSNPSDTTNSINPQFKVTNTGSSAIDLSKLTLRYYYTVDGQKDQTFWCDHAAIIGSNGSYNGITSNVKGTFVKMSSSTNNADTYLEISFTGGTLEPGAHVQIQGRFAKNDWSNYTQSNDYSFKSASQFVEWDQVTAYLNGVLVWGKEPGGSVVPSTQPVTTPPATTKPPATTKPPATTIPPSDDPNAIKIKVDTVNAKPGDTVNIPVRFSGIPSKGIANCDFVYSYDPNVLEIIEIKPGELIVDPNPDKSFDTAVYPDRKIIVFLFAEDSGTGAYAITKDGVFATIVAKVKSGAPNGLSVIKFVEVGGFANNDLVEQRTQFFDGGVNVGDTTVPTTPTTPVTTPTDDSNAVRIKVDTVNAKPGDTVRIPVRFSGIPSKGIANCDFVYSYDPNVLEIIEIEPGDIIVDPNPDKSFDTAVYPDRKIIVFLFAEDSGTGAYAITKDGVFATIVAKVKSGAPNGLSVIKFVEVGGFANNDLVEQKTQFFDGGVNVGDTTEPATPTTPVTTPTTTDDLDAVRIKVDTVNAKPGDTVRIPVRFSGIPSKGIANCDFVYSYDPNVLEIIEIEPGDIIVDPNPDKSFDTAVYPDRKIIVFLFAEDSGTGAYAITKDGVFATIVAKVKSGAPNGLSVIKFVEVGGFANNDLVEQKTQFFDGGVNVGDTTEPATPTTPVTTPTTTDDLDAVRIKVDTVNAKPGDTVRIPVRFSGIPSKGIANCDFVYSYDPNVLEIIEIEPGDIIVDPNPDKSFDTAVYPDRKIIVFLFAEDSGTGAYAITKDGVFATIVAKVKEGAPNGLSVIKFVEVGGFANNDLVEQKTQFFDGGVNVGDTTEPATPTTPVTTPTTTDDLDAVRIKVDTVNAKPGDTVRIPVRFSGIPSKGIANCDFVYSYDPNVLEIIEIEPGELIVDPNPTKSFDTAVYPDRKMIVFLFAEDSGTGAYAITEDGVFATIVAKVKSGAPNGLSVIKFVEVGGFANNDLVEQKTQFFDGGVNVGDTTEPATPTTPVTTPTTTDDLDAVRIKVDTVNAKPGDTVRIPVRFSGIPSKGIANCDFVYSYDPNVLEIIEIEPGDIIVDPNPDKSFDTAVYPDRKIIVFLFAEDSGTGAYAITKDGVFATIVAKVKEGAPNGLSVIKFVEVGGFANNDLVEQKTQFFDGGVNVGDTTVPTTSPTTTPPEPTITPNKLTLKIGRAEGRPGDTVEIPVNLYGVPQKGIASGDFVVSYDPNVLEIIEIEPGELIVDPNPTKSFDTAVYPDRKMIVFLFAEDSGTGAYAITEDGVFATIVAKVKEGAPEGFSAIEISEFGAFADNDLVEVETDLINGGVLVGAPGFSSISAHHHHHHHH |

**Table S3: Primers and cloning strategy used in this study**

|  | **Forward primer** | **Reverse Primer** | **Cloning strategy** |
| --- | --- | --- | --- |
| **ScaA** (*R. champanellensis*) | ttatcaCCATGGgccaccatcaccatcaccataagaagtttatttctgctctg | aatcagCTCGAGttaagctacctcagcgcccag | Restriction using NcoI and XhoI enzymes for insertion in pET28a |
| **X** module from **ScaA** (*R. champanellensis*) | ttatcaCCATGGgccaccatcaccatcaccatgctggtgaattggagttcactt | ttatcaCTCGAGttatgcagcatcaccaacaac | Restriction using NcoI and XhoI enzymes for insertion in pET28a |
| **ScaH** (*R. champanellensis*) | tgacgtTCATGAcacaccatcaccatcaccatgagcaggatccgaagtatat | tgcatgCTCGAGttagcccaccatggactgtg | Restriction using BspHI and XhoI enzymes for insertion in pET28a |
| **SGNH** module **from ScaH** (*R. champanellensis*) | tgacgtTCATGAcacaccatcaccatcaccatgagcaggatccgaagtatat | ttagtcCTCGAGttaccccaggctgttgagaatc | Restriction using BspHI and XhoI enzymes for insertion in pET28a |
| **CipA-ΔXD** | gattaatggcggtgttctggGAGCTCacaaaccggttattgaaggc | gccttcaataaccggtttgtGAGCTCccagaacaccgccattaatc | Primers to introduce a SacI site 5’-of the X-module (see method section for cloning strategy) |
| **Cel8A_S458A** | gacggtgcgattaac**G**cctctgacatgactat | atagtcatgtcagagg**C**gttaatcgcaccgtc | introduction of a mutation in the dockerin S458A |
| **Cel8A_S459A** | ggtgcgattaactcc**G**ctgacatgactatat | atatagtcatgtcag**C**ggagttaatcgcacc | introduction of a mutation in the dockerin S459A |
| **51b SDM:BglII** | cggcgtagaggatcgAGATCTatctcgatcccgcga | tcgcgggatcgagatAGATCTcgatcctctacgccg | Primers used to introduce a BglII site into pET51 |
