# Supplementary figures and images for: Mapping the deformability of natural and designed cellulosomes in solution
Source: Biotechnol Biofuels Bioprod. 2022 Jun 20;15:68. doi: 10.1186/s13068-022-02165-3 (PMC9210761; doi:10.1186/s13068-022-02165-3)

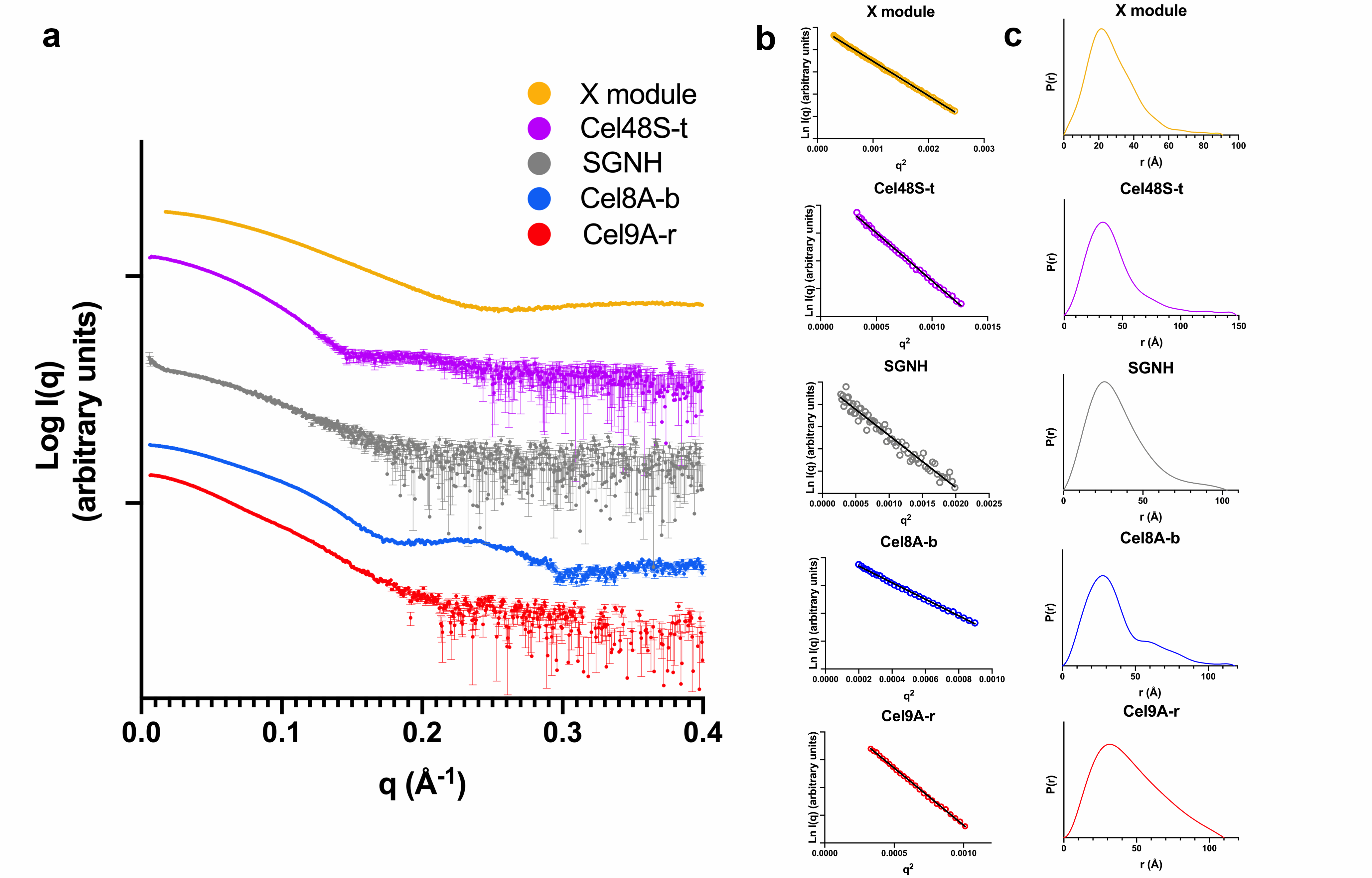

Supplement: Supplementary file 2 — Additional file 2: Figure S1. Experimental SAXS data of the individual modules X and SGNH, as well as the enzymes Cel48S-t, Cel8A-b and Cel9A-r. a. Experimental scattering curves; the color codes are given in the legend. b. Representation of the linear Guinier regions; experimental points are given as open circles (colors as in a) and the black line represents the Guinier-approximation c. Representation of the Fourier-transform P(r)-function for each of the modules and enzymes (colors as in a). See Figure 1 and additional Table S1 for terminology. [file 13068_2022_2165_MOESM2_ESM.tif]

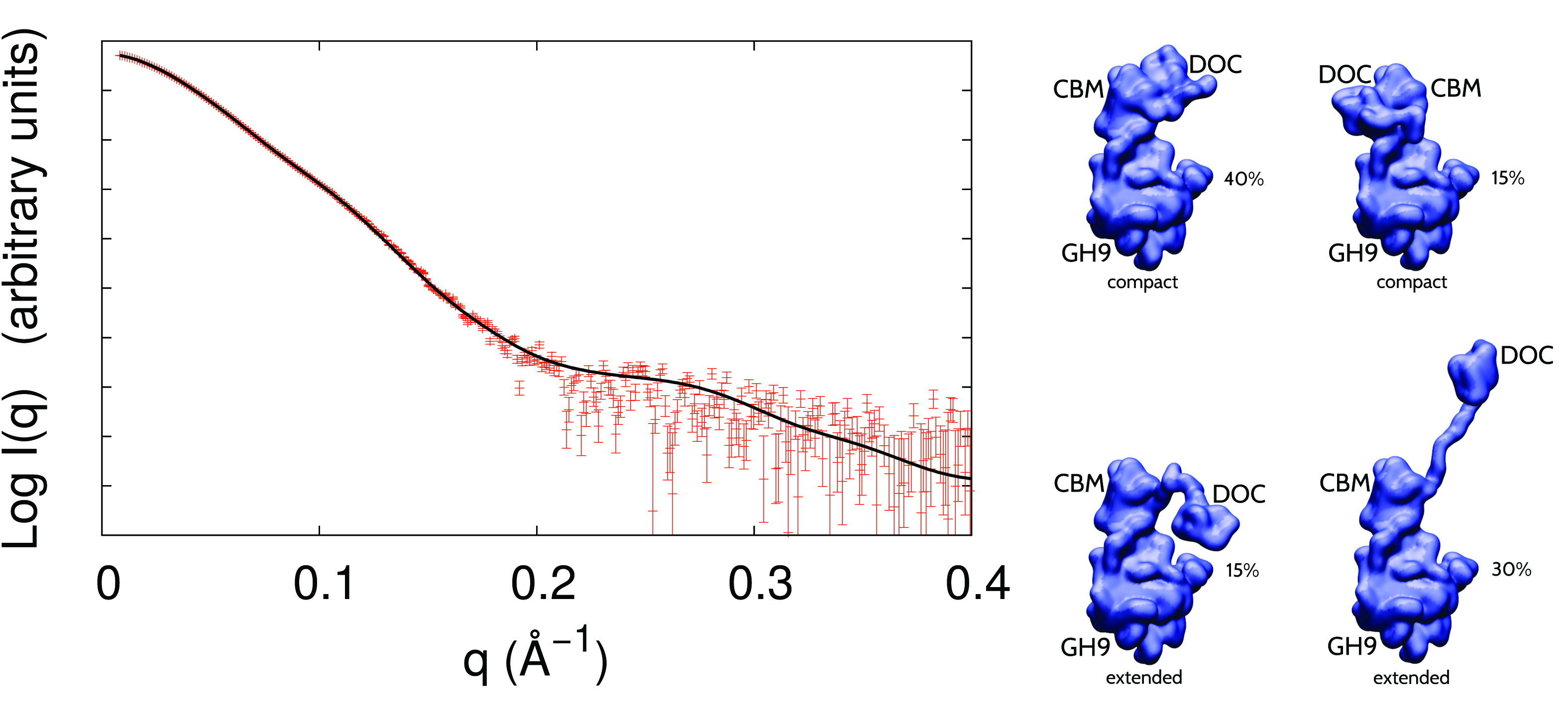

Supplement: Supplementary file 3 — Additional file 3: Figure S2. Left panel: Experimental scattering curve (red points) of Cel9A-r from R. champanellensis and the best fit obtained by a mixture of the structural models obtained by MD-simulations (black line). Right panel: snapshots of Cel9A-r structures obtained by MD-simulations and that best fit the experimental curve with the given proportions (percentage as indicated in the image). The models are represented in blue and the modules composing the protein are indicated as GH9 (catalytic module of Cel9A-r), CBM (CBM3-domain of Cel9A-r) and DOC (dockerin of Cel9A-r). [file 13068_2022_2165_MOESM3_ESM.tif]

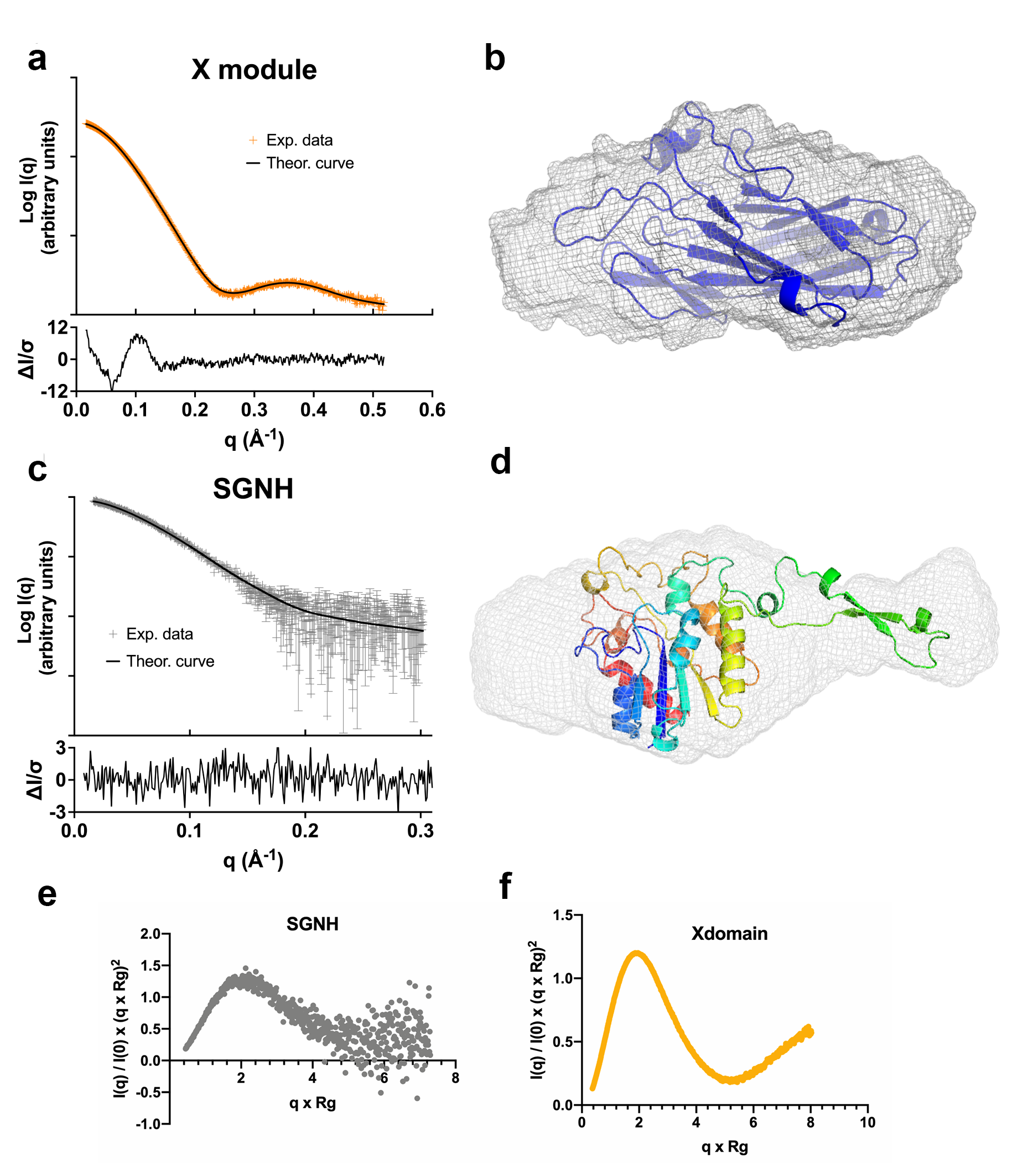

Supplement: Supplementary file 4 — Additional file 4: Figure S3. GASBOR/DAMMIN-Fit and “solution structure” images of the individual modules X and SGNH a. X-module; experimental curve fitted by GASBOR [73]; b. superimposition of the homology model onto one of the most representative GASBOR envelopes. c. SGNH experimental curve fitted by DAMMIN [43]; d. superimposition of the homology model onto the most representative DAMMIN envelope. e. Kratky plot of the scattering curve of the X-module. f. Kratky plot of the scattering curve of the SGNH module. [file 13068_2022_2165_MOESM4_ESM.tif]

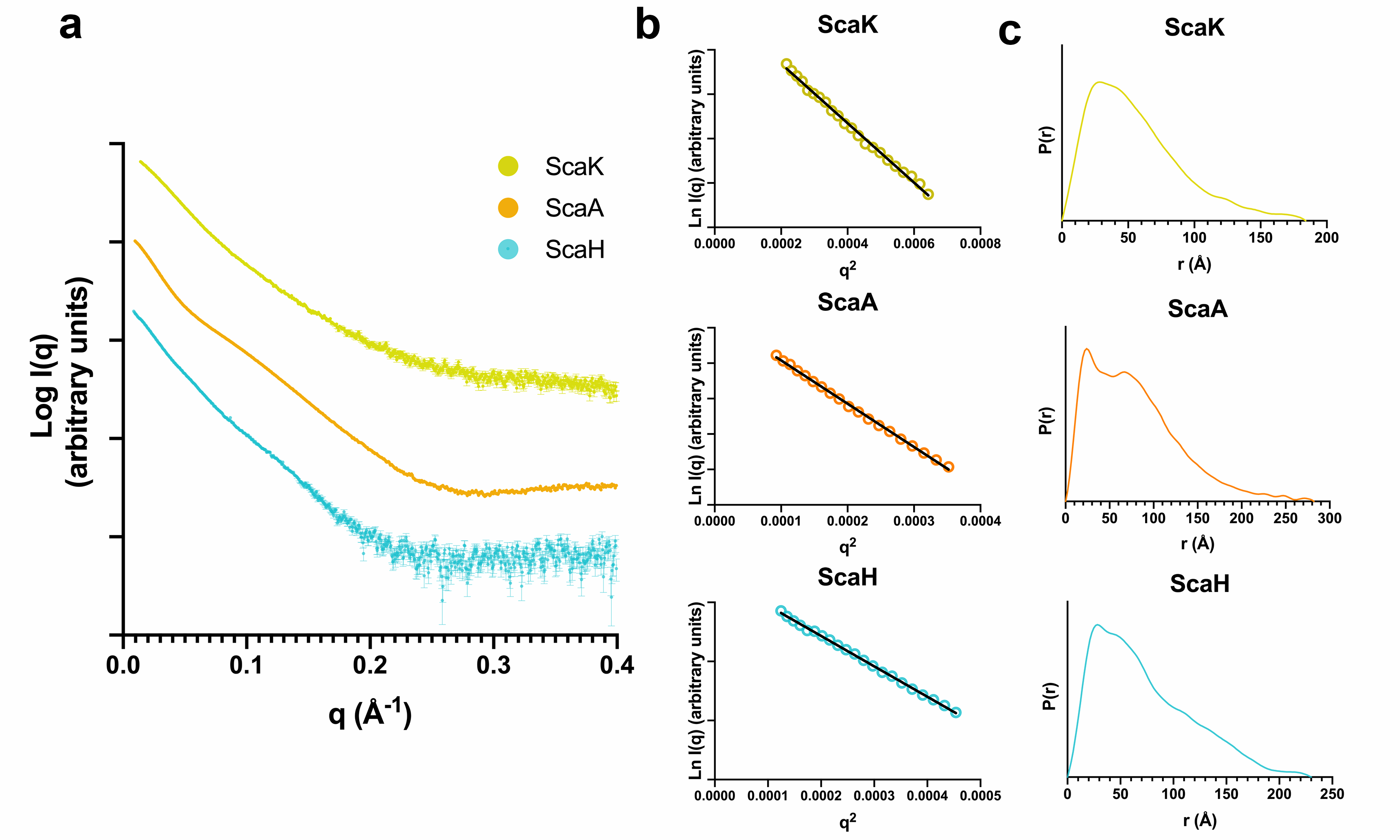

Supplement: Supplementary file 5 — Additional file 5: Figure S4. Experimental SAXS data of the various wild-type ruminococcal Sca-proteins (ScaA, ScaH and SkaK). a. Experimental scattering curves; the color codes are given in the legend. b. Representation of the linear Guinier regions; experimental points are given as open circles (colors as in a) and the black line represents the Guinier-approximation c. Representation of the Fourier-transform, P(r)-function, for each of the scaffoldin proteins (colors as in a). See Figure 1 and additional Table S1 for terminology. [file 13068_2022_2165_MOESM5_ESM.tif]

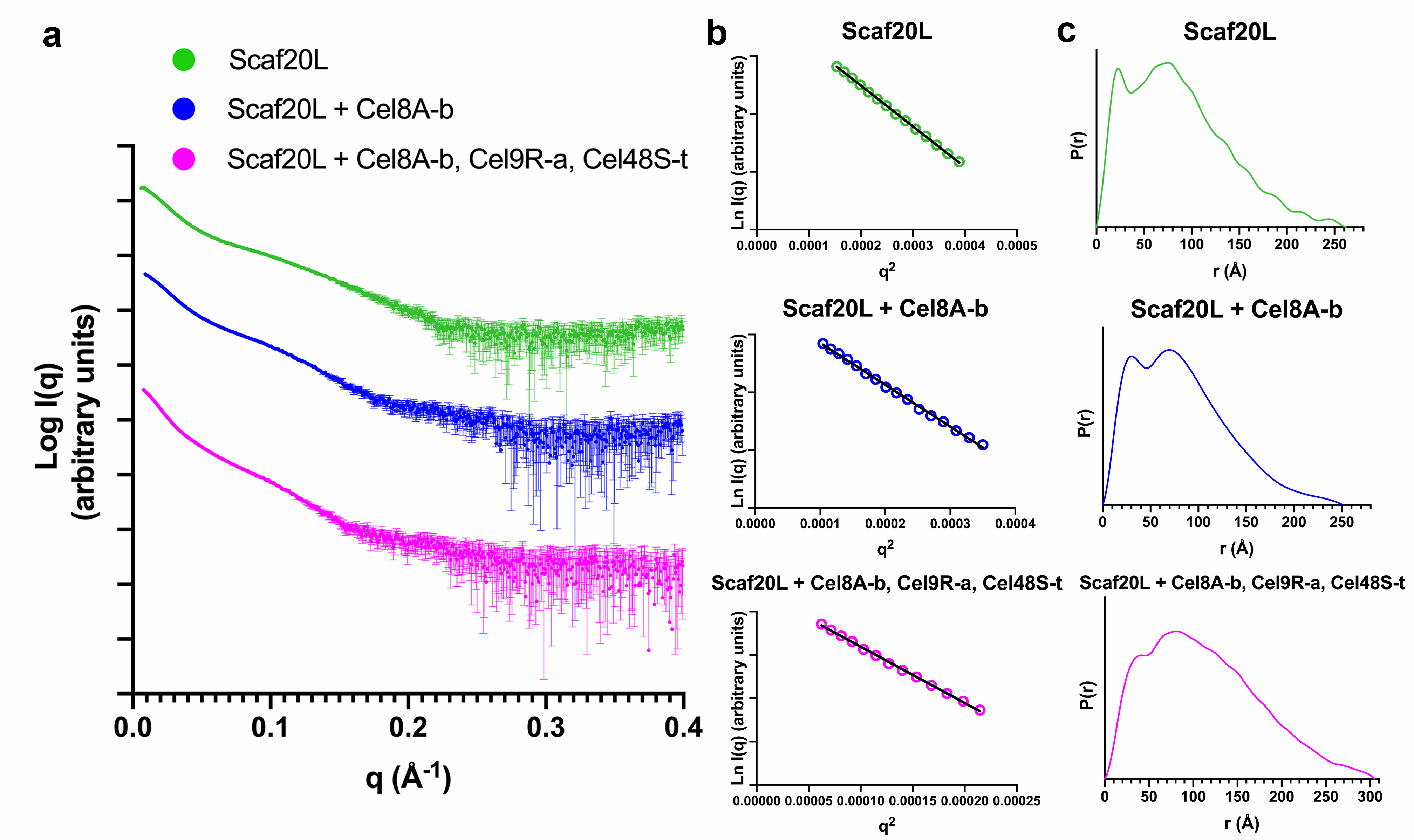

Supplement: Supplementary file 6 — Additional file 6: Figure S5. Experimental SAXS data of the various DCs based on Scaf20L. a. Experimental scattering curves of Scaf20L alone, Scaf20L in complex with Cel8A-b and finally Scaf20L in complex with Cel8A-b, Cel9R-a and Cel48S-t; the color codes are given in the legend. b. Representation of the linear Guinier regions; experimental points are given as open circles (colors as in a) and the black line represents the Guinier-approximation c. Representation of the Fourier-transform, P(r)-function, for each of the DC protein and its complexes (colors as in a). See Figure 1 and additional Table S1 for terminology. [file 13068_2022_2165_MOESM6_ESM.tif]

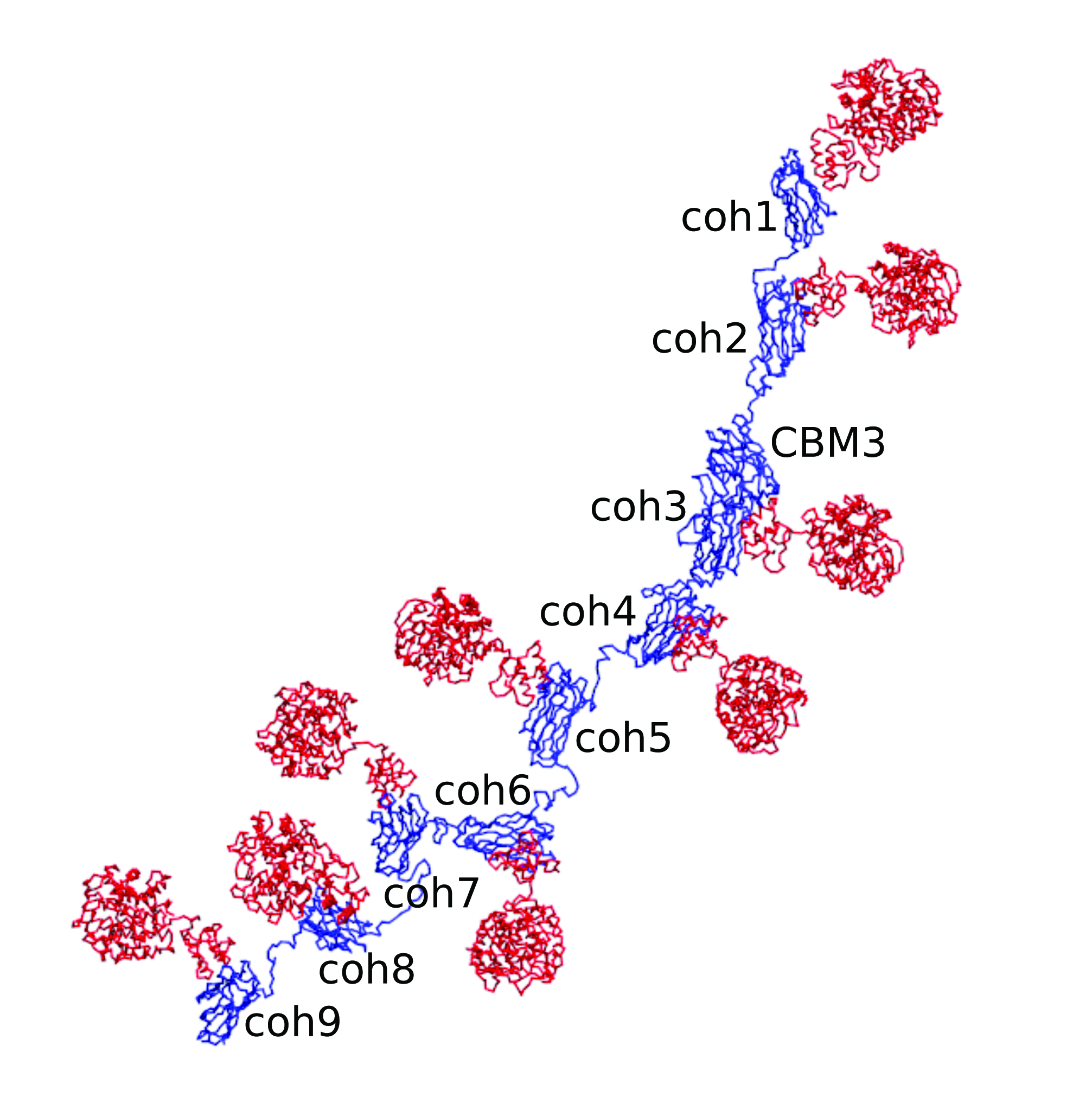

Supplement: Supplementary file 8 — Additional file 8: Figure S7. Cα-trace representation of a single conformational model of CipA in complex with 9 Cel8A-t enzymes, obtained by coarse-grain molecular modeling. [file 13068_2022_2165_MOESM8_ESM.tif]

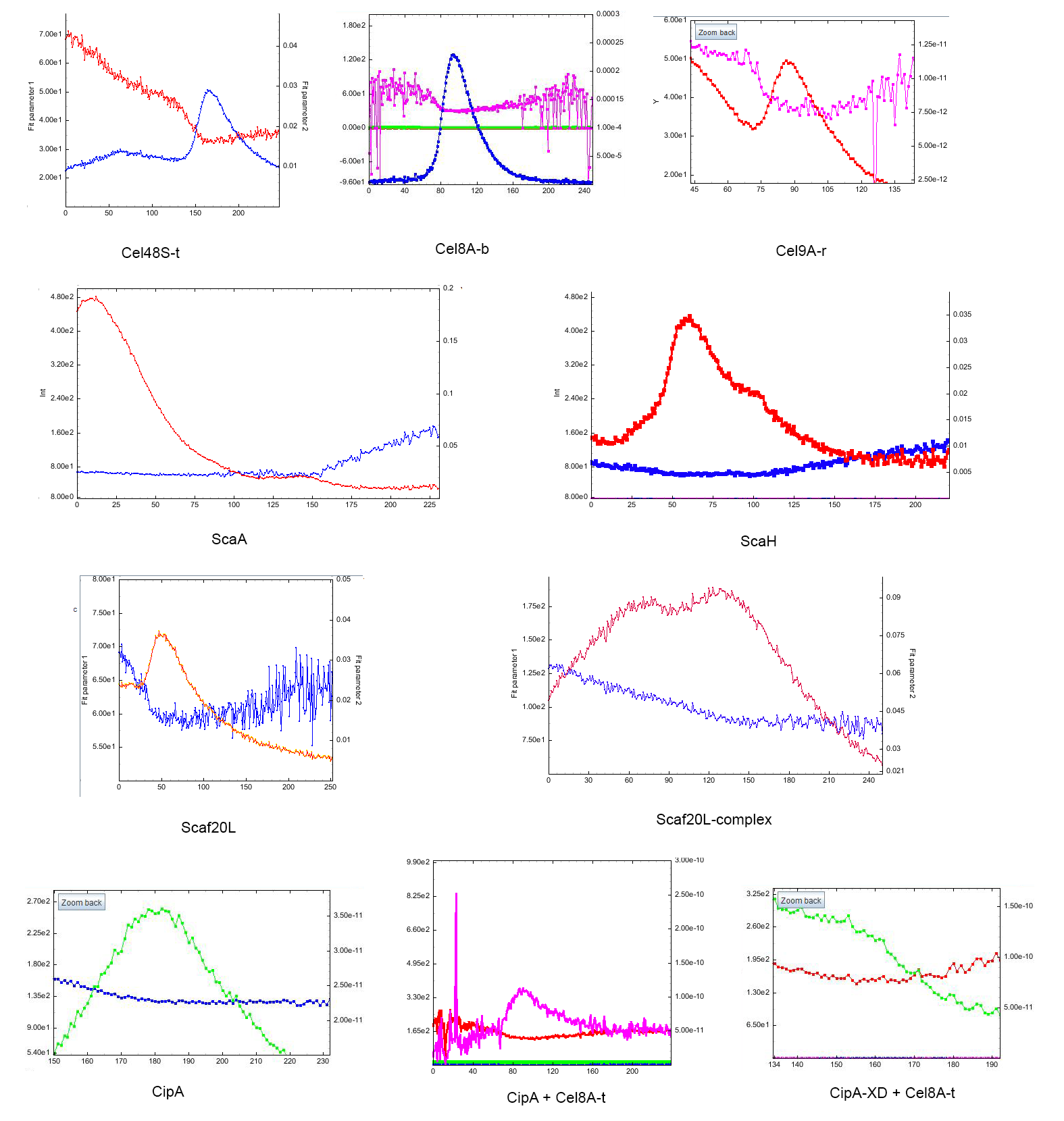

Supplement: Supplementary file 9 — Additional file 9: Figure S8. Experimental SEC–SAXS elution profiles of the major components described in the article, showing I0 vs. Rg values for collected frames. For each data set only images with stable Rg values were averaged to obtain the experimental scattering curve as follows: Cel48S-t image range 160 to 185; Cel8A-b image range 80 to 110; Cel9A-r image range 85 to 105; ScaA image range 2 to 80; ScaH image range 50 to 95; Scaf20L alone image range 65 to 100; Scaf20L in complex with Cel8A, Cel9R and Cel48S image range 150 to 210; CipA image range 180 to 210; CipA+Cel8A-t image range 75 to 115; CipA-ΔXD+Cel8A-t image range 150 to 175. [file 13068_2022_2165_MOESM9_ESM.tif]
